# Nanotechnology and Matrix Metalloproteinases in Cancer Diagnosis and Treatment

**DOI:** 10.3389/fmolb.2022.918789

**Published:** 2022-06-01

**Authors:** Georgina Gonzalez-Avila, Bettina Sommer, A. Armando García-Hernandez, Carlos Ramos, Edgar Flores-Soto

**Affiliations:** ^1^ Laboratorio Oncología Biomédica, Instituto Nacional de Enfermedades Respiratorias “Ismael Cosío Villegas”, Ciudad de México, Mexico; ^2^ Departamento de Investigación en Hiperreactividad Bronquial, Instituto Nacional de Enfermedades Respiratorias “Ismael Cosío Villegas”, Ciudad de México, Mexico; ^3^ Departamento de Investigación en Fibrosis Pulmonar, Instituto Nacional de Enfermedades Respiratorias “Ismael Cosío Villegas”, Ciudad de México, Mexico; ^4^ Departamento de Farmacología, Facultad de Medicina, Universidad Nacional Autónoma de México, Ciudad de México, Mexico

**Keywords:** cancer, matrix metalloproteinases, metastasis nanoparticles, nanotechnology, nanotheranostic

## Abstract

Cancer is still one of the leading causes of death worldwide. This great mortality is due to its late diagnosis when the disease is already at advanced stages. Although the efforts made to develop more effective treatments, around 90% of cancer deaths are due to metastasis that confers a systemic character to the disease. Likewise, matrix metalloproteinases (MMPs) are endopeptidases that participate in all the events of the metastatic process. MMPs’ augmented concentrations and an increased enzymatic activity have been considered bad prognosis markers of the disease. Therefore, synthetic inhibitors have been created to block MMPs’ enzymatic activity. However, they have been ineffective in addition to causing considerable side effects. On the other hand, nanotechnology offers the opportunity to formulate therapeutic agents that can act directly on a target cell, avoiding side effects and improving the diagnosis, follow-up, and treatment of cancer. The goal of the present review is to discuss novel nanotechnological strategies in which MMPs are used with theranostic purposes and as therapeutic targets to control cancer progression.

## Introduction

In accordance with the International Agency for Research on Cancer (IARC), 19.3 million new cases were reported, and the mortality reached 9.9 million individuals in 2020 (Ferlay et al., 2021). Cancer generally does not show clinical manifestations during its early stages and, when diagnosed, metastatic lesions are already present transforming the disease from circumscribed to systemic, becoming the main cause of patients’ demise (Dillekås et al., 2019). Furthermore, when the primary tumor is detected and surgically removed, neoplastic cells with metastatic potential have already been released into the blood or lymphatic circulation to colonize a new organ (Chemi et al., 2021). Unfortunately, the new tumor mass will not be detectable until it is large enough to be identified by conventional diagnostic techniques.

On the other hand, several molecules that participate in tumor evolution have been considered biomarkers to establish an early diagnosis and evaluate cancer progression. Among such molecules, matrix metalloproteinases (MMPs) play a paramount role (Gonzalez-Avila et al., 2019). The expression of MMPs is one of the characteristics necessary for neoplastic cells to detach from the primary tumor and start their journey until they establish a metastatic lesion. Moreover, MMPs are involved in every event of the metastatic process; therefore, their overexpression and enzymatic activity have been considered indicators of tumors’ aggressiveness. For this reason, MMPs have been considered markers during the diagnosis and monitoring of cancer dissemination.

Since metastases cause most cancer deaths, new strategies need to be developed to detect and treat them before the onset of medical signs improving cancer prognosis. In this regard, nanotechnology has a proven potential to control cancer spreading among many novel approaches. Nanoscience explores molecules and structures at a nanoscale range (1–100 nm), including their physical, biophysical, chemical, and biochemical attributes, and their interactions with inorganic and biological systems (Kinnear et al., 2017). Thus, the data obtained through nanoscience allow the manipulation of the particles’ qualities, such as shape, size, and physicochemical characteristics facilitating the design of materials, structures, devices, and systems applicable to diverse activities like industry, agriculture, electronics, and medicine (Bayda et al., 2019). Furthermore, the emergence of nanomedicine has been possible due to the conformation and dimension of nanoparticles (NPs) that can interact with cells even in the intracellular environment, facilitating the management of their pharmacokinetics and distribution, favoring an early diagnosis and therapy of different diseases including cancer (Kinnear et al., 2017; Pelaz et al., 2017).

In this context, nanotechnology has been applied in imaging, biosensing tests, and therapy, avoiding side effects and improving cancer patients’ quality of life. Moreover, nanotechnology has increased the efficiency and sensitivity of detection methods to measure very low quantities of molecules (proteins, DNA, or mRNA) in patients’ samples, promoting their use as biomarkers.

Likewise, since MMPs are related to cancer progression, they can be monitored by techniques improved by nanotechnology to quantify their concentrations and enzymatic activity. Furthermore, MMPs can be therapeutic targets employing specific nanoplatforms in an attempt to control the disease.

Herein we include a brief overview of MMPs’ molecular features and their participation in the metastatic process, particularly in the early steps developed in the tumor microenvironment (TME). MMPs’ relevance in metastasis is highlighted to appraise their potential in cancer diagnosis and treatment and their possible use as therapeutic targets. Subsequently, the application of NPs to improve cancer diagnosis and treatment is examined. Some aspects that must be considered to deliver a nanoplatform to the tumor tissue are included. Finally, how MMPs can be monitored by techniques improved by nanotechnology, the manipulation of MMPs’ characteristics in the design of nanoplatforms for cancer follow-up and treatment, and MMPs’ use as targets for nanotherapies are discussed.

Special emphasis is given to explain how these new strategies took advantage of MMPs’ enzymatic properties to fulfill novel theranostic purposes.

## Molecular Characteristics of the Matrix Metalloproteinases’ Family

MMPs are a group of endopeptidases that require Ca^2+^ and Zn^2+^ ions for their enzymatic activity. At the moment, 28 MMPs have been recognized in vertebrates, but only 24 have been detected in humans (Jackson et al., 2010; Gonzalez-Avila et al., 2019). Interestingly, two MMP-23 forms, MMP-23A and MMP-23B, each one encoded by a different gene, have been identified (Löffek et al., 2011; Galea et al., 2014). However, MMP-23A has been considered a pseudogene (Galea et al., 2014).

**FIGURE 1:**
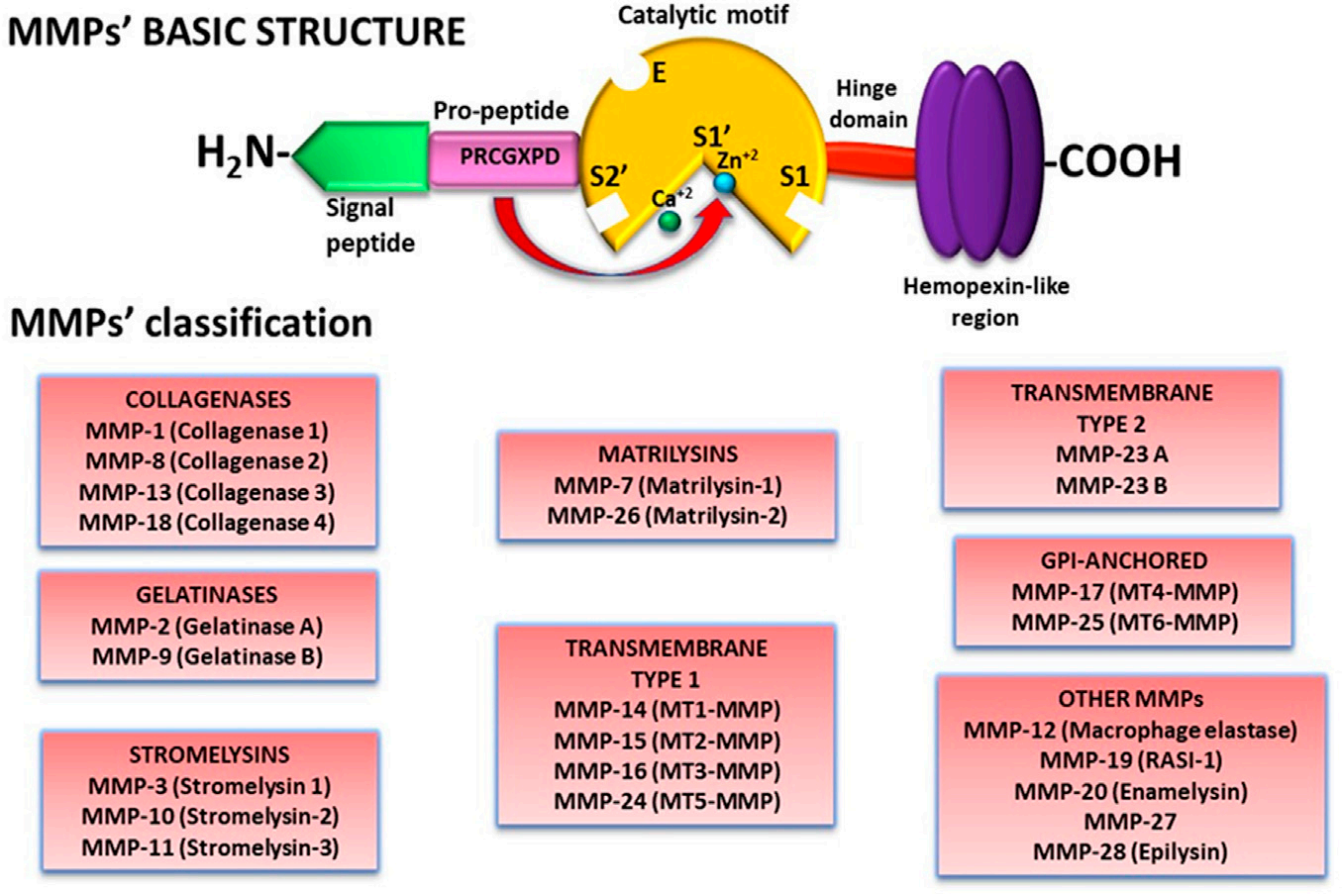
MMPs structure and classification. Besides a basic structure, MMPs have molecular characteristics that differentiate one from another. Therefore, MMPs have been classified according to their substrate specificity and molecular structure in 8 groups. *Abbreviations: MMP, matrix metalloproteinase.*

Almost all MMPs have a similar molecular structure comprised of: 1) a signal peptide in the N-terminal motif that guides MMPs to the secretory pathways or, in the case of the membrane type-MMPs (MT-MMPs), to the cell membrane in which they remain anchored; 2) a pro-peptide that contains the amino acid sequence PRCGXPD enclosing a cysteine residue that includes a thiol (SH-) group that forms a link with a Zn^2+^ ion from the catalytic region and therefore maintaining the enzyme in a latent form; 3) a catalytic motif with a conserved HEXXHXXGXXH sequence and a Zn^2+^ ion that interacts with the pro-peptide cysteine thiol group; 4) a linker/hinge domain rich in proline residues; and 5) a C-terminal hemopexin-like region that participates in MMPs localization, enzymatic activity regulation, and substrate specificity (Figure 1) (Cui et al., 2017; Gonzalez-Avila et al., 2019). In addition to the basic structure, MMPs have other domains that characterize them (Nguyen et al., 2013; Galea et al., 2014; Cui et al., 2017; Gonzalez-Avila et al., 2019).

Likewise, depending on the type and organization of their domains and their targeted substrate, MMPs are cataloged in matrilysins, collagenases, gelatinases, stromelysins, transmembrane type I, transmembrane type II, GPI-anchored, and other MMPs (Figure 1).

Excluding MMP-23, MMPs are produced as pro-enzymes in which the -SH from the cysteine residue in the pro-peptide PRCGXPD sequence is coordinated with the Zn^2+^ ion from the catalytic site at the HEXXHXXGXXH sequence that includes 3 conserved histidine residues linked to the Zn^2+^ ion (Ra and Parks, 2007). The catalytic motif of pro-MMPs adopts a sphere-like shape with a diameter of 40 Å (Tallant et al., 2010). This conformation restrains the linkage of the substrate with the catalytic cleft until the “cysteine-switch” is disturbed by a pro-peptide conformational change that pulls out the cysteine allowing the interaction of an H_2_O molecule with the Zn^2+^ ion at the catalytic site. The conformational change at the pro-domain can be the result of different mechanisms: 1) the excision of the pro-peptide region by other proteinases like plasmin, trypsin, or other MMPs that leads to the activation of the MMP; 2) binding of substrate to other sites different from the catalytic cleft (exosites) producing a partial allosteric activation; and 3) chemical alteration of the cysteine-SH-Zn link by reactive oxygen species (ROS), or by non-physiological substances like 4-aminophenyl mercuric acetate or sodium dodecyl sulfate. After partial activation, the removal of the pro-domain through autoproteolysis confers total enzymatic activity to the MMP (Ra and Parks, 2007; Löffek et al., 2011). Likewise, the MT-MMPs, MMP-11, MMP-17, MMP-21, MMP-25, and MMP-28 with a furin site localized next to the catalytic motif, can be activated inside the cell by a furin-like convertase (Löffek et al., 2011; Itoh, 2015).

Additionally, pro-MMP-2 and pro-MMP-9 are activated by forming complexes with other MMPs and tissue inhibitors of metalloproteinases (TIMPs) (see below).

MMPs have an essential role in many cellular processes in physiological conditions such as blastocyst implantation, embryogenesis, morphogenesis, bone remodeling, wound healing, angiogenesis, aging, tissue repair, cell proliferation, differentiation, and migration due to their interaction with the extracellular matrix (ECM) and their action on growth factors, cytokines, cell adhesion proteins, chemokines, matrikines, matricriptines, cell receptors, cytoskeletal proteins, clotting factors, and hormones, among others (Amălinei et al., 2010; Tallant et al., 2010; Löffek et al., 2011; Ricard-Blum and Salza, 2014; Alaseem et al., 2019).

Conceivably, the dysregulation of their enzymatic activity can cause pathologies such as fibrosis, cancer, rheumatoid arthritis, emphysema, aortic aneurysm, epidermolysis bullosa, neurodegenerative diseases, inflammation, cardiovascular disease, and gastrointestinal ulcer (Amălinei et al., 2010; Tallant et al., 2010). Therefore, MMPs activity must be carefully controlled.

## Control of Matrix Metalloproteinases’ Enzymatic Activity by Tissue Inhibitors of Metalloproteinases

MMPs’ enzymatic activity must be strictly regulated to avoid tissue damage, as mentioned above. MMPs’ activity is modulated by the specific tissue inhibitors of metalloproteinases, TIMPs (Lambert et al., 2004). Four members constitute the TIMPs group: 1) TIMP-1, a soluble glycosylated protein of about 28 kDa; 2) TIMP-2, a 21 kDa soluble non-glycosylated protein; 3) TIMP-3, a glycosylated protein with a molecular weight of around 24–27 kDa, attached to the ECM and cell surface; and 4) TIMP-4, a 22 kDa soluble non-glycosylated protein (Table 1). TIMPs can block the MMPs enzymatic activity and of other enzymes such as ADAMS (a desintegrin and metalloproteinases) and ADAMTS (a desintegrin and metalloproteinases with thrombospondin motifs) which belong to the same metzincin family as MMPs (Jackson et al., 2010). Most of the TIMPs are inducible proteins, except TIMP-2 which is constitutive.

**TABLE 1:** TIMPs’ characteristics.

Property	TIMP-1	TIMP-2	TIMP-3	TIMP-4
kDa	28	21	24/27	22
Amino acid residues	184	194	188	194
N-glycosylation sites	Asn-30, Asn-79	0	Asn-130	0
MMPs weak inhibition	MMP-14, MMP-15, MMP-16, MMP-19, MMP-24	None	None	None
Pro-MMP interaction	Pro-MMP-9	Pro-MMP-2	Pro-MMP-2/-9	Pro-MMP-2
ADAMS inhibition	ADAM-10	ADAM-12	ADAM-10, ADAM-12, ADAM-17, ADAM-19, ADAM-28, ADAM-33	ADAM-17, ADAM-28, ADAM-33
ADAMTS inhibition	None	None	ADAMTS-1, ADAMTS-2, ADAMTS-4, ADAMTS-5	None

ADAMS, a desintegrin and metalloproteinases; ADAMTS, a desintegrin and metalloproteinases with thrombospondin motifs; MMP, matrix metalloproteinase; TIMP, tissue inhibitor of metalloproteinases.

Interestingly, TIMPs have other functions besides MMPs’ enzymatic activity inhibition. One of them is to participate in the activation of MMPs. For example, TIMP-2 forms an activation complex with pro-MMP-2 and MMP-14 (Itoh, 2015). First, two MMP-14 molecules dimerize through their membrane and hemopexin motifs on the cell surface. Then the TIMP-2 C-terminal zone interacts with the pro-MMP-2 hemopexin domain while its N-terminal motif links to the catalytic site of one MMP-14. The second MMP-14 disrupts the MMP-2 pro-peptide at Asn37Leu38 provoking MMP-2 partial activation. MMP-2 total enzymatic activity is achieved by autoproteolysis of the pro-domain (Itoh, 2015). Likewise, TIMP-1 C-terminal extreme binds to the hemopexin motif of pro-MMP-9, while the catalytic site of an MMP-3 molecule interacts with the N-terminal extreme of TIMP-1, forming the pro-MMP-9/TIMP-1/MMP-3 ternary complex (Ogata, et al., 1995). The presence of other active MMP-3 molecules leads to the dissociation of pro-MMP-9 from the complex exposing it to the pro-peptide cleavage by free MMP-3. MMP-1 can also form a pro-MMP-9/TIMP-1/MMP-1 complex inducing the release of pro-MMP-9 without the activation of this pro-enzyme (Ogata, et al., 1995).

## Matrix Metalloproteinases and Cancer Progression

**FIGURE 2:**
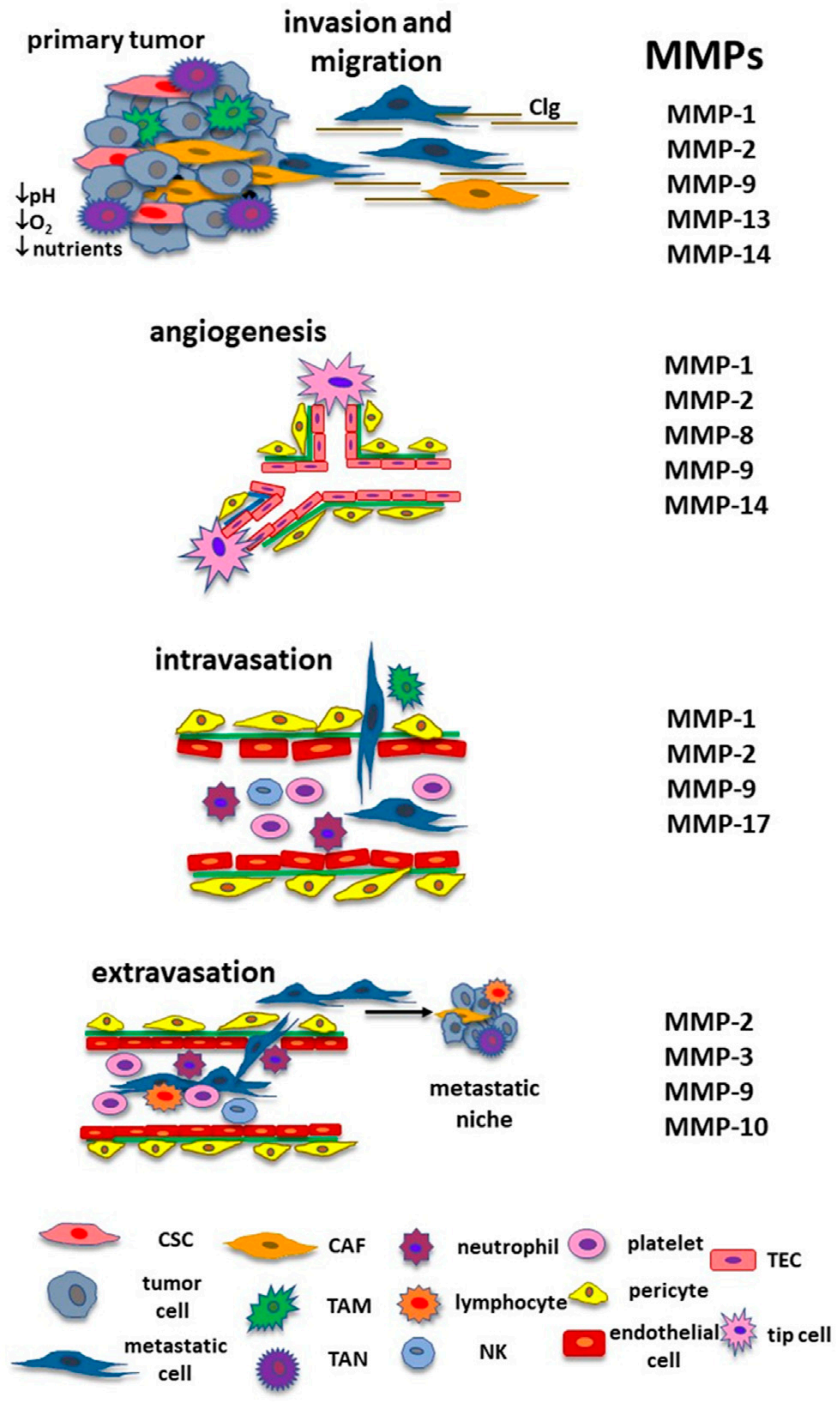
MMPs and the metastatic cascade. During cancer progression, MMPs have a relevant role in each step until they form a new colony in a distant organ. They participate in the remodeling of the ECM and cancer cells’ migration until they reach blood or lymphatic vessels. MMPs are involved in angiogenesis, including the activation of TECs and lymphangiogenesis (MMP-2, MMP-3, MMP-9, MMP-13, MMP-14, and MMP-16, not shown in the figure). MMPs contribute to the intravasation and protect neoplastic cells from immune response and shear stress from the bloodstream. MMPs are also implicated in the extravasation and formation of the metastatic niche. Adapted with permission from Gonzalez-Avila G., Sommer, B., Mendoza-Posada, D.A., Ramos, C., Garcia-Hernandez, A.A., and Falfan-Valencia, R. (2019). Matrix metalloproteinases participation in the metastatic process and their diagnostic and therapeutic applications in cancer. Crit. Rev. Oncology/Hematology 137, 57–83. doi: 10.1016/j.critrevonc.2019.02.010. Copyright 2021. *Abbreviations: CAF, cancer associate fibroblast; CSC, cancer stem cell; ECM, extracellular matrix; MMP, matrix metalloproteinase; NK, natural killer; TAM, tumor associate macrophage, TAN, tumor associated neutrophil; TEC, tumor endothelial cell.*

The origin of tumor cells implies the acquisition of characteristics such as genome instability and mutation, persistent cellular proliferation, evasion of the signals that suppress cell proliferation, apoptosis resistance, immortality, modification of cell metabolism, escape of the immunological surveillance, promotion of inflammation, angiogenesis induction and the activation of mechanisms that lead neoplastic cells to generate metastasis (Hanahan and Weinberg, 2011). However, not all the cells have these characteristics simultaneously, which means that the primary tumor is constituted of a heterogeneous population of cells with diversities in their genetic and phenotypic features and cellular properties (Gerdes et al., 2014). In this context, some tumor cells turn into metastatic initiating cells when they get attributes that lead them to detach from the primary tumor and move to a distant organ (Celià-Terrassa and Kang, 2016). Among these particular properties are: 1) anoikis resistance that prevents cells’ death when they lose cell-cell and cell-ECM interactions; 2) metabolic restructuration as an adaptation to TME conditions such as hypoxia, low nutrient concentrations, and acidic pH; and 3) cell plasticity through the epithelial-mesenchymal transition (EMT) process promoting cell detachment from the primary tumor and invasion to the neighboring tissue (Celià-Terrassa and Kang, 2016). After they detach, metastatic cells move until they reach the blood or lymphatic vessels, and intravasate into the blood or lymphatic circulation, surviving the stream stress and eluding the immunological vigilance. When circulating tumor cells are captured at a capillary bed, they adhere to the endothelial cells and extravasate from the blood circulation to a new organ. Cancer cells regain their epithelial characteristics through the mesenchymal-epithelial transition (MET) process and might adapt to the new microenvironmental conditions generating undetectable micrometastasis that may grow and evolve with the appropriate circumstances (Abdul Pari et al., 2021). However, neoplastic cells can enter a dormant state that can last for many years or be destroyed by the immune system. This chain of events comprises the metastatic cascade (Figure 2).

### Tumor Microenvironment Metabolic Conditions and Matrix Metalloproteinases

The TME is constituted by a diverse cancer cell population, non-structural and structural ECM molecules, cytokines, growth factors, chemokines, and different types of non-neoplastic cells. In this context, MMPs are secreted into the extracellular space, but they have also been identified in mitochondria, cell membranes, nuclei, granules, or cytoplasmic vesicles from neoplastic and non-neoplastic cells (Gonzalez-Avila et al., 2019).

As neoplastic cells proliferate, the conditions of their micro-ecosystem are modified. For instance, solid tumors have zones with a low concentration of O_2_ due to the increase in tumor cell proliferation and the presence of infiltrated immune cells, along with difficulties in the O_2_ delivery caused by a disorganized tumor vasculature and slow blood flow (Rankin and Giaccia, 2016). Furthermore, this hypoxic environment induces a change from aerobic to anaerobic glycolysis in neoplastic cells (Warburg effect) with the consequent increase in lactic acid production and the acidification of the TME (Warburg, 1925). Likewise, the TME acidic pH provokes changes in tumor cells’ morphology and MMPs’ expression; for instance, the formation of tumor cell filopodia and invadopodia with a rise in pro-MMP-2 and cathepsin B in these cell structures (Ji et al., 2019). Interestingly, cathepsin B can activate pro-MMP-2, contributing to the canonical activation pathway that involves the proMMP-2/MMP-14/TIMP-2 activation complex (Kryczka et al., 2019). Moreover, the acidic pH also augments the caveolae, membrane structures that contain cathepsin B binding protein S100A10 and the plasminogen receptor enolase-1 (ENO-1). Besides its effects in the downregulation of the oxidative phosphorylation during the Warburg effect, ENO-1 attaches plasminogen to the urokinase plasminogen/urokinase plasminogen receptor, promoting the production of plasmin involved in the activation of several MMPs including MMP-2 and MMP-9 (Murphy et al., 1999; Hsiao et al., 2013; Capello et al., 2016).

Likewise, low O_2_ levels favor the expression of several genes that supply fitness advantages to cancer cells, including the overexpression of hypoxia-inducible factors (HIFs) (Sullivan and Graham, 2007). HIFs are transcription factors composed of an *a* subunit (HIF-1α, HIF-2α, and HIF-3α) whose expression is controlled by O_2_ levels, and a constitutive *ß* chain (Lu and Kang, 2010).

Hypoxia has been associated with the initial steps of cancer invasion. For example, HIF1α promotes the transcription of Slug, Snail, Twist, and ZEB1 genes involved in the induction of EMT and the inhibition of E-cadherin synthesis (Balamurugan, 2016). Moreover, HIF1-α also controls the expression of genes necessary to maintain cancer stem cells, such as CD133, CD44, Myc, Oct-4, Nanog, and Sox-2 (Balamurugan, 2016). Likewise, HIF-1α promotes other events that initiate in the TME, such as angiogenesis, lymphangiogenesis, and cell migration. Moreover, the hypoxic environment can also prevent the immune response of cytotoxic T and natural killer cells. (Rankin and Giaccia, 2016). In addition, hypoxia affects myeloid-derived suppressor cells and tumor-associated macrophages (TAMs), inducing them to contribute to cancer progression (see below).

Interestingly, the hypoxic TME increases the expression of MMPs. Table 2 shows some MMPs whose transcription is regulated by HIF-1α.

**TABLE 2:** MMPs inducible by HIF-1α.

MMPs	Cancer cells	References
MMP-1	Lung cancer cells	Shyu et al. (2007)
	BmMSCs	Lin et al. (2008)
MMP-2	Human colon carcinoma cells	Krishnamachary et al. (2003)
	Glioma cells	Fujiwara et al. (2007)
	Esophageal cancer cells	Jing et al. (2012)
MMP-3	BmMSCs	Lin et al. (2008)
MMP-9	Glioma cells	Fujiwara et al. (2007)
	Breast cancer cells	Choi et al. (2011)
MMP-13	Nasopharyngeal carcinoma cells	Shan et al. (2018)
	Ovarian cancer cells	Zhang et al. (2019)
MMP-15	Pancreatic cancer cells	Zhu et al. (2011)
	Lung cancer cells	Zhu et al. (2011)

BmMSCs, bone marrow mesenchymal stem cells; MMPs, matrix metalloproteinases.

### Matrix Metalloproteinases Promote Epithelial-Mesenchymal Transition in Cancer Cells

On the other hand, several MMPs stimulate the expression of EMT genes like Snail, Twist, Slug, Zeb1, and Zeb2 that confer mesenchymal characteristics to tumor cells. Additionally, MMPs inhibit the synthesis and disrupt E-cadherin, increase the production of fibronectin, vimentin, and N-cadherin, up-regulate Wnt5a, enhance the Wnt/βcatenin signaling and suppress the expression of epithelial markers like cytokeratin-18 and zonula occludens-1 (Table 3) Additionally, MMP-28 can indirectly stimulate EMT through the induction of TGFβ synthesis and activation (Illman et al., 2006]. Interestingly, molecules involved in EMT can, in turn, stimulate the production of MMPs (Gonzalez-Avila, et al., 2020).

**TABLE 3:** MMPs’ effects on EMT.

MMP	EMT event	References
MMP-2	E-cadherin disruption	Brusgard et al. (2015)
MMP-3	E-cadherin disruption, ↓ E-cadherin expression, ↑ Rac1b, ↑ Wnt3a/β-catenin signaling	Noë et al. (2001)
		Radisky et al. (2005)
		Blavier et al. (2010)
MMP-7	E-cadherin disruption	Noë et al. (2001)
MMP-9	E-cadherin disruption, ↑FBN, ↑ N-cadherin, ↑ Vimentin, ↑Snail	Symowicz et al., 2007
		Lin et al. (2011)
MMP-12	↑E-cadherin disruption, ↑Snail, ↑N-cadherin, ↑FBN	Sei-Jung Lee et al. (2014)
		Hung et al. (2021)
MMP-14	E-cadherin disruption, ↑Wnt5a, ↑FBN, ↑Vimentin, ↑Slug, ↑Snail, ↑Zeb1, ↑Zeb2, ↓E-cadherin, ↓ZO-1, ↓CK-18	Cao et al., 2008
		Yang et al., 2013
		Li et al. (2015)
MMP-20	↑N-cadherin, ↑Vimentin,↑Snail, ↑Twist	Aseervatham and Ogbureke (2020)

CK-18, cytokeratin-18; EMT, epithelial-mesenchymal transition; FBN, fibronectin; MMPs, matrix metalloproteinases; Zeb1, zinc finger E-box binding homeobox 1; ZO-1, zonula occludens-1.

#### Non-Neoplastic Cells: Cancer-Associated Fibroblasts and Tumor-Associated Macrophages

In addition to the TME condition effects on cancer cells’ evolution, non-neoplastic cells can contribute to tumor cells’ metastatic characteristics acquisition. Non-neoplastic cells induce the production of cancer cells’ MMPs but can also respond to the molecules and MMPs released by tumor cells becoming allies in cancer progression (Heneberg, 2016). For instance, cancer-associated fibroblasts (CAFs) that derive from bone marrow-derived mesenchymal stem cells, fibroblasts, adipocytes, endothelial cells, pericytes, epithelial cells, and smooth muscle cells are attracted by chemokines and cytokines released by tumor cells that switch them to CAFs. Then, activated CAFs synthetize MMP-1, MMP-2, MMP-3, MMP-9, MMP-11, MMP-13, MMP-14, and MMP-19 allowing basement membrane and ECM remodeling, angiogenesis, cell proliferation, and immune response evasion (Gaggioli et al., 2007; Kessenbrock et al., 2010; Lederle et al., 2010; Bates et al., 2015; Yamaguchi and Sakai, 2015; Heneberg, 2016).

Other examples of non-neoplastic cells transformed by cancer cells are TAMs. TAMs originate from the blood circulating monocytes mobilized to the tumor by cytokines secreted by stromal and cancer cells (Bonavita et al., 2015). According to O_2_ concentrations, monocytes can be polarized into TAMs-M1 with cytotoxic functions when localized in areas with normal O_2_ levels or into TAMs-M2 in hypoxic zones (Bonavita et al., 2015). Moreover, growth factors and cytokines from the TME such as TGFβ, colony stimulating factor, IL-4, and IL-10 stimulate TAMs-M2 polarization (Sica et al., 2015). Furthermore, TAMs-M2 can synthetize MMP-1, MMP-9, MMP-11, and MMP-12 allowing these cells to participate in the ECM turnover and angiogenesis (Pettersen et al., 2011; Fujimura et al., 2017; Pelekanou et al., 2018]. In addition, TAMs-M2 induce the expression and release of MMP-1, MMP-3, MMP-10, and MMP-14 by the effect of IL-1β and the synthesis of MMP-9 through the stimulation of TNFα on the neoplastic cells (Kamoshida et al., 2013; Whipple, 2015).

Besides CAFs and TAMs, other cells respond to neoplastic cell signals, including MMPs, to evade the immunologic surveillance or secreted cytokines, chemokines or MMPs that, in turn, induce tumor cells proliferation, decrease apoptosis, and favor invasion and angiogenesis. Such is the case of tumor-associated neutrophils, mast cells, and cancer-associate adipocytes (Gonzalez-Avila et al., 2020).

Therefore, MMPs from the neoplastic cells or non-neoplastic cells participate in the induction of the events necessary to transform a tumor cell into a cell capable of detaching from the primary tumor and migrating to a distant tissue. Figure 2 shows MMPs’ participation in each step of the metastatic cascade. More information regarding MMPs’ role in the TME and the metastatic cascade has been compiled in two previous works from our group (Gonzalez-Avila et al., 2019; Gonzalez-Avila et al., 2020).

## Nanomedicine

### Nanomaterials Used in Cancer Treatment

Multiple inorganic and organic nanomaterials have been successfully employed in the diagnosis, follow-up, and treatment of cancer. NPs possess singular characteristics that allow manipulation of their size, form, composition, physicochemical properties, and surface functionalization, conferring them the ability to interact with different organs, cells, cell organelles, proteins, lipids, and DNA. On the other hand, when using nanomaterials (NMs) for biomedical purposes, their toxicity, biodistribution, inflammatory response, the type of interactions with cells, cellular endocytosis rates, and the route of administration, whether oral, dermal, inhaled or more frequently intravenous, should be considered (Anselmo and Mitragotri, 2016; Kinnear et al., 2017).

**FIGURE 3:**
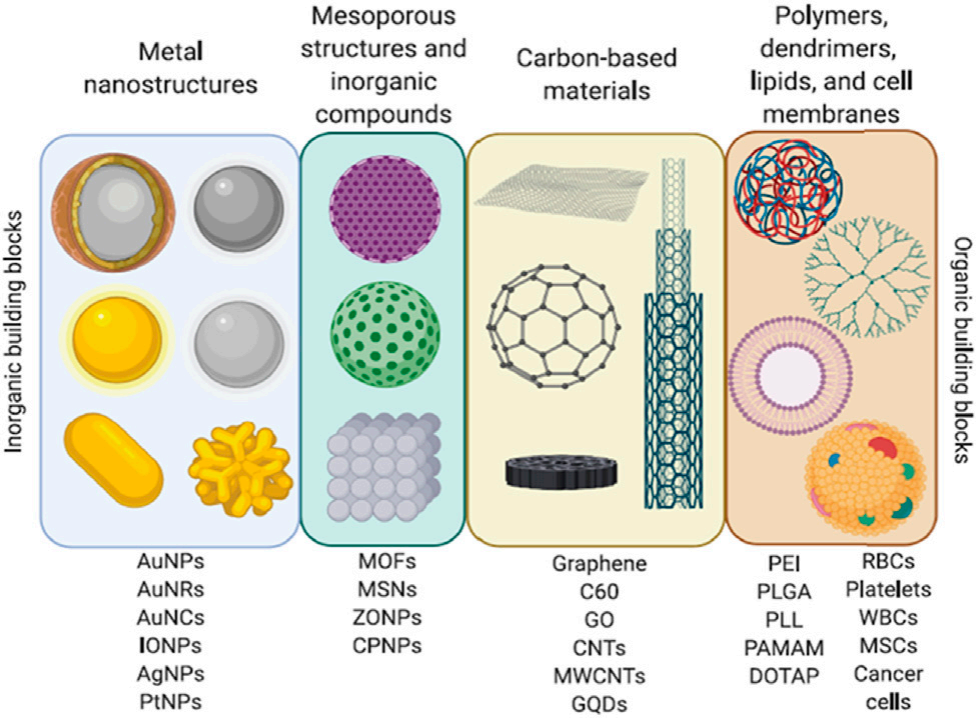
Nanomaterials. Diverse organic and inorganic nanomaterials are used to construct nanosystems for biomedical applications. Adapted with permission from Seaberg J, Montazerian H, Hossen MN, Bhattacharya R, Khademhosseini A, Mukherjee P. Hybrid Nanosystems for Biomedical Applications. ACS Nano. 2021 February 23;15(2):2099–2142. doi: 10.1021/acsnano.0c09382. Copyright 2021 American Chemical Society.

Nowadays, the most frequently NMs used in nanomedicine are: 1) metal NPs and their oxides (gold, gold nanoclusters [GNCS/AuNCs], titanium, zinc, zinc oxide, thallium, platinum, silver, silica, iron oxide [IO], quantum dots [QDs], superparamagnetic iron oxide NPs [SPIONs], and upconverting NPs [UCNPs]); 2) carbon-based nanomaterials (carbon nanotubes [CNTs], fullerenes, nanodiamonds [NDs] graphene oxide [GO]); 3) organic NPs (dendrimers, ferritin, lipid-based NPs, micelles, and polymers); and 4) hybrid systems that have been created by combining organic and inorganic NPs (Figure 3) (Pelaz et al., 2017; Khan et al., 2018; Lee et al., 2021; Seaberg et al., 2021). Inorganic NPs have been employed in imaging techniques and biosensors. In contrast, NMs such as liposomes, lipid micelles, polymers, dendrimers, CNTs, CO, NDs, ribonucleic acid NPs (RNPs), and protein constructs have been used mainly in vaccines and drug delivery systems (Anselmo and Mitragotri, 2016; Pelaz et al., 2017; Khan et al., 2018; Lee et al., 2021; Seaberg et al., 2021). Likewise, in the design of inorganic/organic hybrid systems, the properties of both types of molecules are considered. For example, inorganic NPs like AuNPs, iron oxide NPs (IONPs), zinc oxide NPs (ZONPs), mesoporous silica NPs (MSNs), and graphene QDs (GQDs) can be photo-responsive, conductive, superparamagnetic, pH-responsive, catalytic, and photoluminescent. Similarly, organic NMs properties to be considered in the construction of hybrid systems are the response to stimuli such as pH, temperature and redox, biocompatibility, transmembrane delivery ability, cell stability, high loading function, solubility, nontoxicity, and stimuli/targeting ligands (Lee et al., 2021; Seaberg et al., 2021). Some examples of organic nanomaterials that have been used in hybrid systems are dendrimers (poly-amidoamine [PAMAM]), polymers (poly-lactic-co-glycolic acid [PLGA], chitosan, poly-L-lysine [PLL]), liposomes, micelles, and cells membranes from white blood cells (WBCs), red blood cells (RBCs), cancer cells and mesenchymal stem cells (MSCs) (Figure 3). Interestingly, using membranes from cancer cells to cover NPs increases their directionality towards a specific target (Seaberg et al., 2021). Likewise, hybrid systems structure consists of an inorganic NPs, polymer or liposome core, encapsulated by a shell constituted by liposomes, dendrimers, polymers, cell membranes, inorganic NPs, or inorganic nanoclusters such as GNCs. In addition, various molecules like antibodies, nucleic acids, hydrophilic polymers, targeting ligands, proteins, and/or phospholipids can be attached to the surface of the shell. These systems are used in drug delivery, gene therapy, phototherapy (PTT), and photodynamic therapy (PDT) (Seaberg et al., 2021).

### Nanocarriers and Cancer

Nanotechnology has been considered a promising therapeutic tool to control cancer since NPs, due to their physicochemical characteristics, can deliver a drug to a specific target increasing its circulating half-life and conserving its stability and solubility, reducing side effects. (Shi et al., 2017). Moreover, NPs increase therapeutic molecule (DNA, mRNA, siRNA, and proteins) accumulation and tumor penetration, cellular internalization, and intracellular release. Furthermore, a nanocarrier can also facilitate drug transit through epithelial-endothelial surfaces like the blood-brain barrier. Likewise, some NPs such as AuNPs, GO, and IONPs have therapeutic effects.

On the other hand, the most frequent route of nanotreatments administration is intravenous, which offers advantages and disadvantages. For instance, NPs administrated intravenously can be delivered to the tumor by passive, active, or both mechanisms. Passive targeting takes advantage of the pathophysiological characteristics of the solid tumor vasculature associated with the disorganized and leaky tumor vascularization caused by the angiogenesis process and poor lymphatic drainage that increases permeability and retention of macromolecules, including NPs. Therefore, due to the enhanced permeability and retention (EPR) effect, large NPs can accumulate and remain in tumor tissue (Shi et al., 2017). Moreover, the EPR effect allows the delivery of NPs conjugated with cytotoxic drugs to tumor tissue reducing toxic effects. However, the EPR effect may vary from patient to patient, type of tumor, and in the same tumor over time since angiogenesis is a dynamic process constantly changing the tumor vasculature. Likewise, inflammatory molecules and pro-angiogenic factors (IL-1β, IL-2, IL-6, proteases [MMP-9], and vascular endothelial growth factors [VEGFs]) can enhance the EPR effect (Wu, 2021). In addition, characteristics such as size, shape (filomicelles, cylindrical, ellipsoidal, or discoidal), and negative or neutral surface charges must be considered while designing NPs to exploit EPR effect therapeutic benefits in cancer treatment. Interestingly, the collagen content of the capillary wall, blood MMP-9/TIMP-1 ratio, and quantification of angiogenic factors such as fibroblast growth factor 2, VEGF A, and MMP-9 have been considered predictive biomarkers of the EPR effect (Shi et al., 2017).

Likewise, to improve NPs delivery to neoplastic cells, active targeting has been developed consisting of the functionalization of NPs with specific tumor ligands such as antibodies or peptides that can bind to specific molecules at the cancer cells’ surface. Once the NP is attached to the cell, it is internalized by receptor-mediated endocytosis, and its cargo is released due to the intracellular acidic pH or enzymes’ activity (Yu et al., 2021).

On the other hand, when NPs enter the circulatory system, their surface interacts with different ions, molecules, and cells. Of particular interest is the interaction between NPs surface and plasma proteins such as albumin, immunoglobulins, apolipoproteins, and fibrinogen (de Puig et al., 2017). Proteins form a corona whose composition depends on the NPs formulation, shape, size, surface charge, functional groups, and the protein affinity for the NPs surface composition. Therefore, since protein coronas are one of the most relevant factors that can alter NP properties affecting their biodistribution, toxicity, cellular uptake, and intracellular trafficking, conjugation of NPs surface with serum proteins such as albumin has been considered as an alternative to improve delivery to a specific target and cell intake (de Puig et al., 2017). Moreover, NPs functionalization with amino, hydroxyl, carboxyl groups, or PEG reduces toxic effects and augments cellular intake. Furthermore, PEG and other polymers can decrease non-specific binding, augment targeting to specific cell receptors and prevent the removal of NPs by the mononuclear phagocyte system.

Additionally, the effectiveness of NPs as nanocarriers depends on several factors, for instance, their circulation time in blood, target cell specificity, efficiency in tumor penetration and accumulation (EPR effect), cellular internalization of coated NPs (with PEG, for example), and intracellular release of the pharmaceutical agent in response to an internal (i.e., pH, oxidative stress or intracellular enzymes), or external stimulus such as near-infrared (NIR) light (Parodi et al., 2020).

Likewise, one of the recent contributions of nanotechnology to cancer treatment is the generation of nanoplatforms with theranostic purposes. The theranostic approach includes the possibility of diagnosis, disease evolution follow-up, treatment, monitoring of drug release, and biodistribution, reducing invasiveness and toxic side effects. In this regard, different chemotherapy drugs have been integrated into nanoplatforms; such is the case for paclitaxel, doxorubicin (DOX), oxaliplatin, cisplatin, docetaxel (DTX/DOC), vincristine sulfate, methotrexate (MTX), and rapamycin (Shi et al., 2017). Interestingly, miRNA and siRNA can also be delivered by nanocarriers in cancer therapy. Moreover, the nanotheranostic system can include radiotherapy, PTT, PDT, or chemotherapy in its design. In addition, the NPs employed for nanotheranostic purposes may be inorganic (copper, CNTs, NDs, gold, NMPs, graphene, IONPs, MSNs, QDs, silver, and zinc), organic (nanogeles, polymeric NPs, liposomes, dendrimer, solid lipid NPs, nanocrystals, multifunctional micelles, and nanoemulsions), or hybrid (gold-iron NPs, polymer-lipid nanocomplex, gold-silica NPs, polymeric-metal NPs). Besides, NPs’ surfaces can be conjugated to specific ligands like transferrin, EGFR, folic acid, hyaluronic acid, or monoclonal antibodies against tumor cell markers to drive the nanotheranostic system to a specific tissue. Furthermore, the nanoplatform can also include responsive elements to TME to facilitate therapeutic agents release into the tumor. Moreover, the platform can be combined with diagnostic and monitoring techniques such as CT, MRI, ultrasound imaging, PET, SPECT, photoacoustic (PA), and live-cell imaging (Paliwal et al., 2020). Importantly crucial is the notion that the NPs used in the theranostic systems must be efficiently degraded and depurated from the body after fulfilling their purpose.

## Matrix Metalloproteinases’ Contribution to Cancer Diagnosis and Treatment

Because of the relevant participation of MMPs in cancer progression, they can be considered markers and therapeutic targets for cancer. Moreover, several nanoplatforms use MMPs’ enzymatic activity to deliver and release cytotoxic drugs to tumor tissue.

### Matrix Metalloproteinases and Nanodiagnosis by Biosensors

MMPs’ concentrations and enzymatic activity have been evaluated in cancer patients’ tissue and body fluids samples. However, liquid biopsy (LB) is preferable as a less invasive method for early diagnosis, treatment response evaluation, and disease progression monitoring. In this context, the methods frequently used to quantify MMPs’ concentrations and their enzymatic activity are ELISA, chemiluminescence immunoassay, immunohistochemistry, western blot, and zymography in LB and tissue samples. However, for the detection of small MMPs’ amounts, strategies based on nanotechnology have been developed. Some biosensors built using nanotechnology to quantify small concentrations and the enzymatic activity of MMPs, are outlined below.

For example, a biosensor constructed using gelatin crosslinked to Fe(II) chelating alginate NPs was developed to detect MMP-2 and MMP-9 gelatinase activity in urine samples (Acharya et al., 2017). The Fe(II) is used as the catalyst agent of a Fenton’s reaction in this system. Fe(II) is turned off in the biosensor due to its conjugation with the alginate polymer. Furthermore, the assembly of gelatin to Fe(II)-alginate NPs promotes the formation of aggregates that precipitate, and when samples containing active MMP-2 or MMP-9 are added, gelatin is degraded, and floating NPs are equivalent to the amount of active MMPs. Then FE(II) is released from the alginate NPs with acid, and becomes ready to participate in a Fenton’s reaction when the chromophore is added. In addition, collagenase type IV was used to determine the assay limit of detection (LOD), which was 1 pg/ml. Moreover, this assay was tested in a pilot study for the diagnosis of bladder cancer in which the sensitivity was 100%, specificity 85%, and a negative predictive value of 100%.

On the other hand, most of the biosensors employed to determine MMPs concentrations and enzymatic activity include in their design a peptide cleavable by these enzymes, while the rest of the biosensor components favor the stability and cleavage kinetic. Some of the MMP cleavable sequences used in biosensors and drug carriers are listed by Xiong, J. and Gao, H. (Xiong and Gao, 2017). For example, the detection of active MMP-2 can be achieved using a biosensor constituted by photoluminescent QDs conjugated with a biotin-peptide GPLGVRGK with a susceptible site LG**↓**VR to be cleaved by MMP-2 (arrow) and a black hole quencher (BHQ) (Pillai et al., 2017). Streptavidin functionalized to QDs (CdSe/ZnS core/shell structure) can bind to biotin-peptide to form the QD-(pep-BHQ-1) nanoprobe in a quencher state. Moreover, this system is based on the Föster resonance energy transfer (FRET), in which QD is the energy donor and the biotin-pep-BHQ-1 is the acceptor material. When active MMP-2 is incubated with the nanoprobe, it cuts the peptide, and the QD separates from the BHQ emitting photoluminescence. By using this nanosystem, active MMP-2 concentrations of about 1 ng/ml can be measured in LB samples.

Additionally, other materials such as UCNPs and metal nanoclusters have been used to detect active MMPs in FRET biosensors. For instance, a nanosystem was designed, including upconversion phosphors (UCPs) as energy donors and carbon nanoparticles (CNPs) as energy acceptors. In this nanoprobe, CNPs act as an effective quencher of UCPs particles photoluminescence produced during their excitation when NIR light is used (Wang et al., 2012). Moreover, this FRET biosensor includes the peptide GHHYYGPLGVRGC with the sequence PLG↓VR that is cleaved by MMP-2 (arrow) and the amino acid sequence HHYY that links the peptide to the UCPs’ surfaces. As a result, the UCP-peptide-CNP nanosystem can detect as low as 10 pg/ml of active MMP-2 in plasma samples.

Similarly, a nanocomplex that includes fluorescent AuNCs, GO as the quencher (energy acceptor), mercaptoundecanoic acid (MUA) that stabilizes AuNC structure, and the MMP-9 sensitive peptide GPLGMSRGLC has been constructed for the detection of MMP-9 enzymatic activity (Nguyen et al., 2017). In this system, when active MMP-9 cuts the peptide, the interaction between the GO quencher and AUNCs is disturbed, allowing fluorescence emission with a LOD of 2.5 ng/ml. Moreover, the sensitivity of the peptide/MUA/AuNC/GO nanocomplex in biological systems was confirmed when used to determine active MMP-9 concentrations in culture medium from human breast cancer MCF-7 cells after incubation with phorbol 12-myristate 13-acetate (PMA) that increases MMP-9 expression. The experiment results showed an increase of active MMP-9 concentrations of 3.2 ng/ml, 5.03 ng/ml, and 11.8 ng/ml at 4, 8, and 12 h, respectively, after incubation with PMA.

Likewise, MMP-7 has been quantified by surface-enhanced Raman spectroscopy (SERS) immunoassay in blood from pancreatic cancer patients (Granger et al., 2013). For this purpose, primary antibodies against MMP-7 were immobilized by AuNPs on a glass slide, and then the serum samples were added to separate and capture the antigen. Finally, the extrinsic Raman label (ERL) suspension that consists of AuNPs functionalized with the Raman reporter 5,5′-dithiobis [succinimidyl-2-nitrobenzoate (DSNB) and the primary antibody was added. Then, MMP-7 concentrations were calculated by a Raman spectroscopy using the average relative intensity of DSNB. The LOD for MMP-7 using SERS immunoassay was 2.28 ng/ml. Furthermore, the results obtained by SERS were compared with values obtained by ELISA, in which the LOD for MMP-7 was 31.8 ng/ml. Moreover, less sample volume was required for SERS analysis (∼2.5 µL/well) than for ELISA assay (∼20 µL/well) in MMP-7 quantification.

Additionally, an optical biosensor to detect MMP-2 and MMP-7 enzymatic activity was developed with a multiplex fluorescence system that uses lanthanide-doped UCNPs (Cao et al., 2018). The nanoprobe include the peptides (His)6GPLGVRGK-TAMRA and (H)6VPLSLTMGK-FITC that contain the cleavage sites for MMP-2 and MMP-7, respectively, The UCNPs were constructed in the core/shell structure NaYF4:Gd^3+^/Yb^3+^@NaYF_4_:Yb^3+^/Tm^3+^/Er^3+^. Tm^3+^, and Er^3+^ are doped in the shell layer to improve their luminescence emission. Moreover, the peptide-TAMRA and peptide-FITC were linked to UCNPs’ surfaces through their poly-histidine tails. TAMRA and FITC work like quenchers of Eu^3+^ green and Tm^3+^ blue emissions, respectively. When active MMP-2 and MMP-7 are added to the system, they cut their respective peptides allowing quencher dissociation and luminescence emission. This biosensor was used to quantify the enzymatic activity of MMP-2 and MMP-7 in the medium of human fibrosarcoma HT1080 and K562 cells and serum from healthy subjects. The LOD for MMP-2 was 2.2 ng/ml, while the LOD for MMP-7 was 13.9 ng/ml.

On the other hand, a nanomechanical biosensor to measure active MMP-2 has been developed using a cantilever biosensor that detects molecular processes on its surface, producing a change in its deflection fluctuations or resonant frequency (Choi et al., 2017)., The biosensor surface was modified with 3-aminopropyltrimethoxysilane and functionalized with PEGylated peptides linked to the sequence GPLGVRGK that contains the cleavage site for MMP-2. The biosensor is installed in a fluid cantilever holder that quantifies MMP-2 in small amounts of fluid samples. When MMP-2 cuts the peptide, an increase in the biosensor resonant frequency occurs due to changes in the mass of the functionalized peptide on the biosensor surface in which the VRGK peptide remains linked to it while the GPLG peptide is released. Moreover, the kinetic rate of proteolysis depends on the total mass of the peptide chains cleaved by MMP-2.

The nanomechanical biosensor has been used to quantify active MMP-2 and proteolysis rate in media from lung cancer A549, H460, and H322 cells and human fetal lung cancer WI-26 (control). The total mass of peptides cleaved by MMP-2 was ∼300 pg in H460 cells, and ∼200 pg in A549 and H322 cell media. No secreted active MMP-2 was observed in the medium from the control cells. Furthermore, active MMP-2 was measured in blood from mice transplanted with lung cancer H460 cells at stage I and stage II of the disease. The total mass of cleaved peptide and the kinetic rate of proteolysis for stage I were 148 pg and 6.12 × 10^–2^/min, respectively, while for stage II they were 548 pg and 13.07 × 10^–2^/min. In addition, using the nanomechanical biosensor, active MMP-2 was determined in blood samples from lung cancer patients with stage IV-M1a and IV-M1b (Choi et al., 2017). Results showed that both, the mass of cleaved peptide chains and the kinetic rate of proteolysis, were higher in patients with stage IV-M1b lung cancer than in stage IV-M1a (348 pg and 7.7 × 10^–2^/min, and 226 pg and 5.58 × 10^–2^/min, respectively).

**FIGURE 4:**
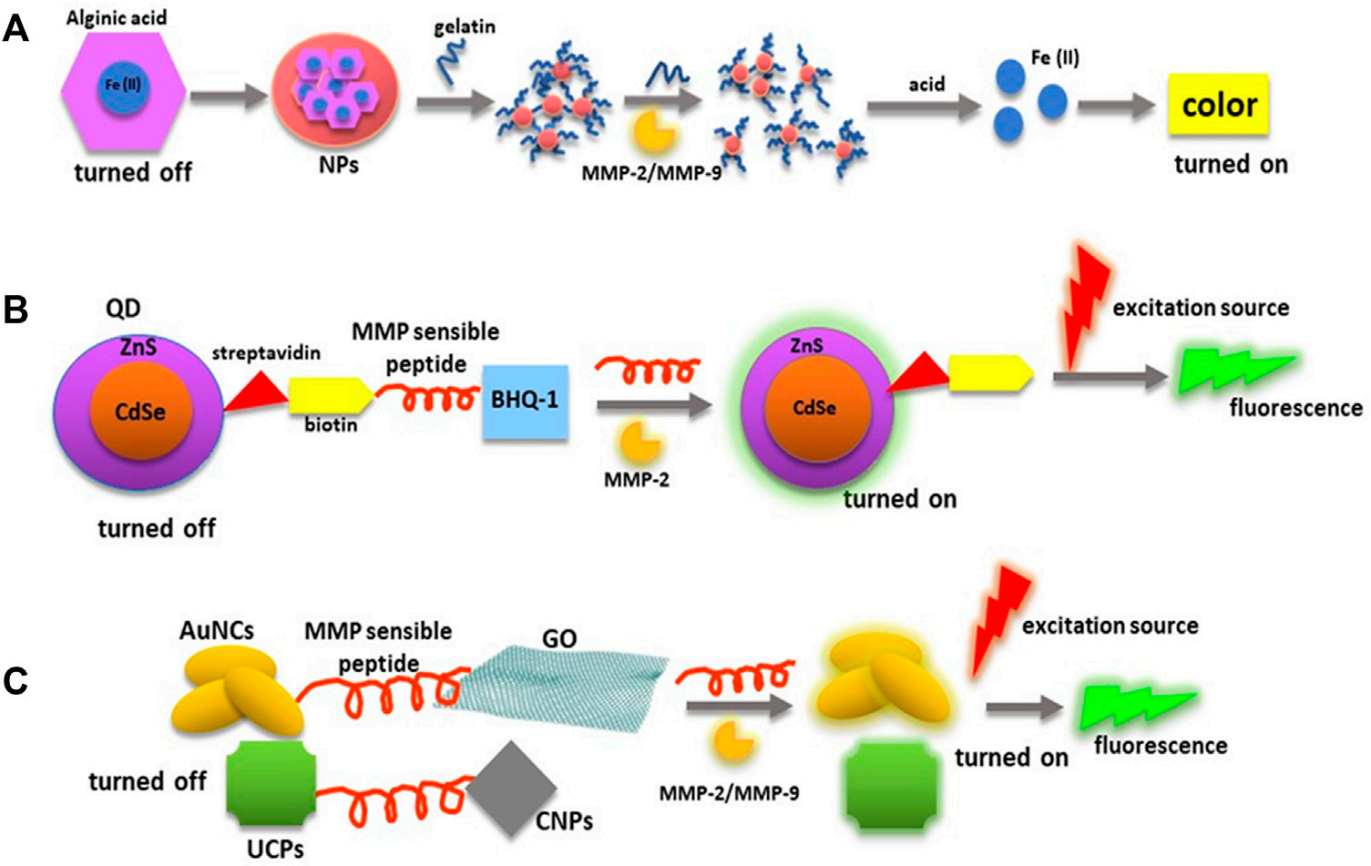
MMPs’ and diagnostic biosensors. MMPs’ substrates have been incorporated in nanoprobes to quantify MMPs’ enzymatic activity. **(A)** This panel shows a nanocarrier involved in the quantification of MMPs’ enzymatic activity through a Fenton’s reaction in which gelatin is used as MMP-2/MMP-9 substrate. **(B)** Peptides degradable by MMPs can be used as a bridge between an energy donor (QDs) and as an energy acceptor (BHQ-1) to turn off fluorescence emission until the peptide is cleaved by an MMP. **(C)** MMP cleavable peptides can be directly linked to fluorescent NPs (AuNCs and UCPs) and NMs employed with quencher functions (GO and AuNCs). *Abbreviations: AuNCs, gold nanoclusters; BHQ-1, black hole quencher-1; CNPs, carbon nanoparticles; MMP, matrix metalloproteinase; NMs, nanomaterials; NPs, nanoparticles; QDs, quantum dots; UCPs, upconversion phosphors.*

Based on the characteristics of nanobiosensors aforementioned, Figure 4 presents some examples in which nanotechnology is used to measure MMPs’ levels and their enzymatic activity.

### Matrix Metalloproteinases and Imaging Nanotechniques

Imaging techniques have been developed to detect tumor cells that overexpress MMPs, incorporating probes with peptides degradable by them. For instance, a nanosystem that includes the sensitive peptide RSCitG-HPhe-YLY cleaved between the Gly and homophenylalanine (HPhe) by MMP-14 was designed for the detection and guiding surgery of glioma tumors using near-infrared fluorescence (NIRF) and PET images (Kasten et al., 2020). The peptide is linked to the NIRF IR dye800 and its quencher IR QC-1 (NIRFdye-substrate peptide-quencher). In addition, ^64^Cu, or ^68^Ga, are attached to a second binding peptide (peptide probe) with the sequence HWKHLHNTKTFL that can bind to MMP-14 for PET visualization of cells expressing MMP-14. This binding peptide is joined to the substrate peptide. When the nanosystem is incubated with cells that have active MMP-14, the disruption of the substrate peptide occurs, the quencher is released, and the peptide probe attaches to MMP-14 in the cell membrane allowing the identification of cells overexpressing MMP-14 by fluorescence emission. Moreover, NIRF signals and radiolabeled localization of tumor cells were possible by NIRF and PET/CT images after the intravenous injection of the nanoprobe in mice bearing glioma tumors.

Interestingly, the InPQDs and the UCNPs systems were joined through the peptide SGAVRWLLTA sensible to MMP-2 activity to create the UCNP-p@InP nanosystem (Chan et al., 2019). The arginine-glycine-aspartic (RGD) acid that can bind to integrin αVβ3 was also integrated into this complex to target cancer cells, increasing the nanocomplex accumulation in tumor tissue. Furthermore, when the (UCNP-p@InP)-RGD nanosystem is irradiated with infrared light, the upconverted energy is transferred to InPQDs, and red light is emitted in the absence of active MMP-2. On the contrary, when active MMP-2 cleaves the peptide, the QDs are released from the system, and the infrared light stimulation of the UCNPs produces green fluorescence emission. This system was probed in oral cancer cells with different metastatic behavior. It is known that tongue squamous cell carcinoma HSC4 cells (high metastatic potential) have more active MMP-2 than gingival squamous cell carcinoma Ca922 cells (low metastatic potential). Therefore, when these cells were incubated with the UCNP-p@InP)-RGD complex and after 808 nm light stimulation, HSC4 emitted green fluorescence from the UCNP, and Ca922 red fluoresce from the QDs. Moreover, to compare both cell lines simultaneously, a mice model was created injecting HSC4, and Ca922 cells in the animal’s left and right thigh, respectively. After tumors grew, the UCNP-p@InP)-RGD complex was intravenously applied, and different zones of fluorescence were observed in which green zones corresponded to areas where acive MMP-2 was present. Notwithstanding, latent MMP-2 might have been present in the red fluorescent areas.

Indeed, imaging techniques used in cancer diagnosis and follow-ups such as MRI, PET, and SPECT have been improved by inserting a peptide cleavable by an MMP. For example, activable cell-penetrating peptides (ACPPs) were included in the design of nanoprobes. ACPPs consist of a PLGLAG peptide with an MMP-2 cleavage site localized between a polyanionic penetrating cell peptide E8 and a polycationic peptide R9 to neutralize the polyanionic segment (Olson et al., 2010). In addition, image monitoring of the nanosystem was obtained by integrating a PANAM dendrimer core conjugated with a fluorescence (Cy5) dye, Gd, or both covalently bound to the polyglutamate site (polyanionic) of the peptide. Moreover, the dendrimer favored the accumulation of the image tracer in the tumor and decreased glomerular filtration enhancing the probe circulation time (Olson et al., 2010). Therefore, when the nanoprobe arrived close to MMPs’ overexpressed cells, active MMP-2 disrupted the PLGLAG peptide, and the polyanionic peptide was released, allowing the interaction of the polycationic peptide with the cell surface inducing its internalization. Furthermore, mice bearing HT1080 tumors were injected with the dendrimer-ACCP (ACCPD) with Gd, Cy5, or both image labels. After 48 h, images were taken demonstrating the accumulation of the Gd and fluorescence in the tumor cells.

**FIGURE 5:**
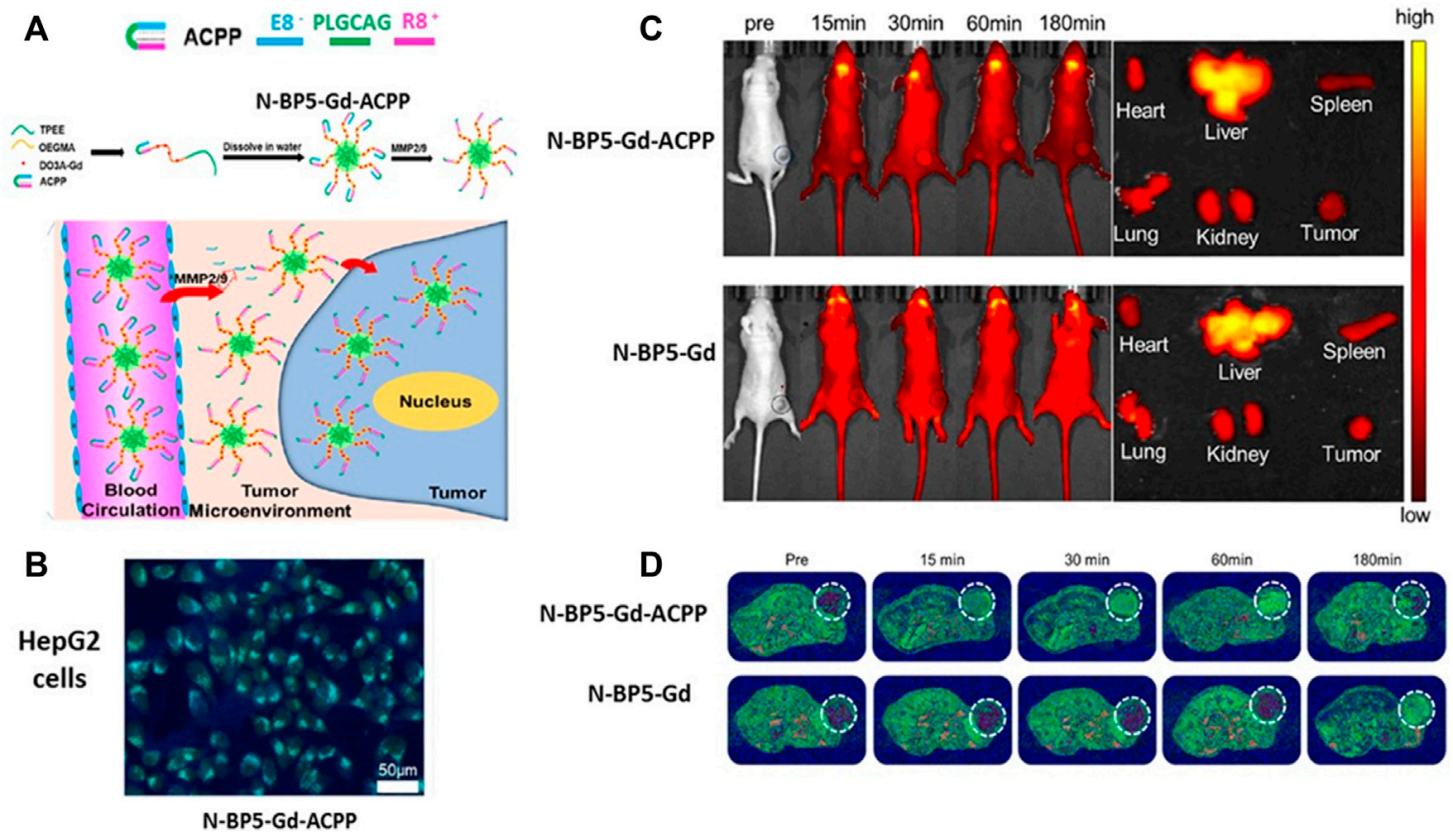
MMPs’ sensible peptides in imaging techniques. **(A)** MMP cleavable peptide included in an activable cell-penetrating peptide (ACPP) was integrated into the N-BP5-Gd-ACPP nanoprobe favoring its cell uptake. **(B)** Fluorescence image of hepatocellular carcinoma HepG2 cells incubated with N-BP5-Gd-ACPP. Incubations with N-BP5-Gd were not visible under the fluorescence microscope (not shown in the figure) **(C)** Fluorescence images show the differences between HepG2 tumor-bearing animals injected with N-BP5-Gd-ACPP or N-BP5-Gd. **(D)** MR images demonstrate the rapid distribution of the N-BP5-Gd-ACPP nanoprobe in the tumors compared to animals injected with N-BP5-Gd. Adapted with permission from Xia, B., Yan, X., Fang, W.W., Chen, S., Jiang, Z., Wang, J., et al. (2020). Activatable Cell-Penetrating Peptide Conjugated Polymeric Nanoparticles with Gd-Chelation and Aggregation-Induced Emission for Bimodal MR and Fluorescence Imaging of Tumors. *ACS Appl. Bio. Mater* 3, 1394–1405. doi: 10.1021/acsabm.9b01049. Copyright 2020 American Chemical Society. *Abbreviations: MMP, matrix metalloproteinase.*

Similarly, ACPPs have been conjugated with Gd-DO3A, oligo (ethylene glycol), methyl ether methacrylate (OEGMA), and AIEgens TPEE (2-[4-vinylphenyl-1,1,2-triyl] tribenzene) that emits strong fluorescence (Xia et al., 2020). This nanosystem, named N-BP5-Gd-ACPPs, has a size of ∼40 nm in which the hydrophobic AIEgens TPEE constitutes the core, hydrophilic copolymers P(OEGMA-co-Gd-DO3A) form the shell, and ACPPs compose the corona. In addition, the peptide used in the ACCP is PLGCAG, which is suitable for cleavage by MMP-2 and MMP-9. Furthermore, the N-BP5-Gd-ACPPs nanoprobe was internalized by hepatocellular carcinoma HepG2 cells when MMP-2 and MMP-9 released in the culture medium cut the peptide included in the ACPP. MR images demonstrated the presence of neoplastic cells in livers from HepG2 tumor-bearing mice 15 min after the injection of the N-BP5-Gd-ACPPs. Moreover, after 7 days of nanosystem postinjection, there was no evidence of damage to the liver, kidney, heart, spleen, or lung (Figure 5).

Besides AIEgens and Cy5, other fluorescent NPs can be conjugated with ACPP, such as UCNPs and QDs, to develop bimodal systems for MR and fluorescence images.

On the other hand, nanosystems detectable by SPECT have also been developed using an MMP cleavable peptide. For instance, two different radionuclides with distinct gamma emission spectrums can be visualized using a nanoprobe that includes a diethylenetriamene pentaacetate (DTPA) labeled with ^64^Cu or ^111^In, a peptide with a cleavage site for active MMP-9, a tyrosine amino acid residue radiolabeled with ^125^I, and a cysteine that links the DPTA-GPLGVRGKGYGAhxC-NH sequence to AuNPs (Black et al., 2015). In addition, PEG is integrated into the NPs to stabilize the NPs in an aqueous solution and enhance blood circulation time. Experiments were conducted in mice injected either with epidermoid carcinoma A431 cells that express high levels of MMP-9, or the luciferase-expressing 4T1Luc mouse breast carcinoma cells that synthesize low MMP-9 levels. After tumors grew, dual radiolabeled (^111^In and ^125^I) AuNPs were injected, and 24 h after SPECT/CT images were performed, showing ^111^In accumulation in both tumors and ^125^I in the thyroid gland. Moreover, standardized uptake values (SUV) obtained from imagen quantification of tumor accumulation of ^111^In demonstrated higher values in A431 than in 4T1Luc tumors. This behavior was probably due to the active MMP-9 from A431 tumors that cleaved the peptide allowing NPs to be up-taken faster by A431 neoplastic cells than 4T1Luc cells. However, 4T1Luc cells accumulated ^111^In signal over time while A431 SUVs decayed, pointing out that the nanoprobe pharmacokinetics is related to MMP-9 expression.

Likewise, the nanoprobe AMP-CNP-DOTA-^64^Cu for PET was created using ^64^Cu radiolabeled glycol chitosan NPs (CNPs), NIR Cy5.5 fluorescence dye, BHQ-3 fluorescence quencher, and MMP substrate peptide GPLGVRGKGG (Lee S. et al., 2014). When MMP-2 or MMP-9 from tumor cells cuts the peptide, NIR Cy5.5 fluorescent dye is dissociated from CNPs, and fluorescence is emitted. Furthermore, the AMP-CNP-DOTA-^64^Cu was used in A549 tumor-bearing mice, and after injection, NIRF/PET images were obtained to see the nanoprobe distribution. The results showed that the NIRF signal was visible in the tumor region 1 h later and reached a plateau at 6 h, while radioactivity was detected 2 h postinjection, reaching a plateau 24 h later. Moreover, the *ex vivo* analysis of several organs and tumors demonstrated that the highest accumulation of NIRF and radiolabel signal was found in the tumor mass. However, the kidney showed an increase in fluorescence, probably due to the clearance of the Cy5.5 dye at 48 h, while the radioactivity signal that traces CNPs distribution was increased in blood, liver, spleen, and kidney 48 h after the nanoprobe injection.

### Matrix Metalloproteinases in Nanocarriers for Nanotheranostic Approach

Nanocarriers for drug delivery have been used instead of free chemotherapeutic agents. In this context, the combination of gemcitabine (GEM) and erlotinib (ERL) conjugated to NPs was used *in vitro* and *in vivo* assays to treat pancreatic cancer (Yin et al., 2020). Briefly, GEM/ERL was bound to PEG-DSPE-maleimide to construct the GEM/ERL-PEG-DSPE-maleimide NPs. Then, these NPs were conjugated through maleimide to the non-substrate MMP-14 sequence MT1-AF7p (HWKHLHNTKTFLC), forming the M-M GEM/ERL NPs (∼167 nm) in which the MT1-AF7p binds to MMP-14 present in neoplastic cell membranes improving cellular uptake of the nanosystem. The large size of these nanodrugs favors tumor accumulation due to the EPR effect together with the functionalization of the NMs with tumor marker ligands such as the non-substrate MMP-14 peptide (active targeting). Moreover, the PEG attachment to the NPs allows their stability in blood circulation until they reach a tumor.

**FIGURE 6:**
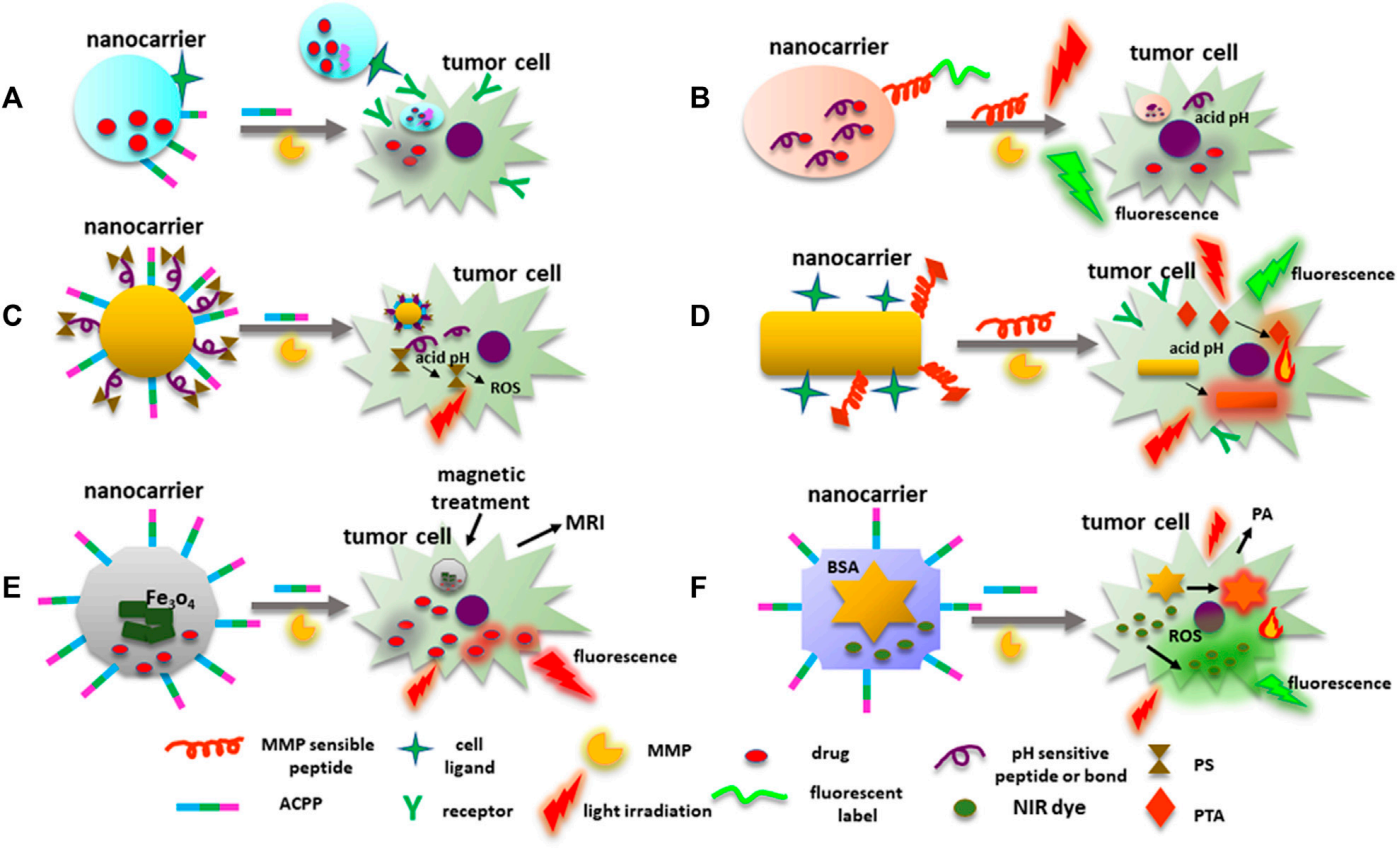
MMPs’ cleavable peptides in nanocarriers’ construction. **(A)** An example of a nanosystem designed for drug transport with ligands to target neoplastic cells. ACPPs with an MMP sensible peptide, are directly conjugated to NM surface to facilitate nanocarrier cell uptake. **(B)** Fluorescence label linked to a nanocarrier surface through an MMPs’ sensible peptide emits fluorescence when active MMPs disrupt the peptide. Drugs transported by the nanocarrier can be linked to the NP through an acidic pH-sensitive bound or peptide that is disrupted in the cell, releasing the cytotoxic drug. **(C)** ACCP-MMP sensible peptide and PSs for PDT can be integrated to a nanosystem. PS is conjugated into the nanoprobe through an acidic pH-sensitive link or peptide. Once the PS is released into the cell, light irradiation induces ROS production damaging the tumor cell. **(D)** Nanocarriers can contain PTAs for PTT attached to the NPs by MMP cleavable peptides, NPs included in the probe can behave as PTAs. PTAs can also emit fluorescence under light irradiation. **(E)** Nanoplatforms used for chemotherapy, and magnetic treatment can also be used for fluorescent and MR images. MMP sensitive peptides are employed to deliver the system to the neoplastic cells. DOX under light irradiation emits red fluorescence. **(F)** Nanocarriers can be used simultaneously for PTT, PDT, and monitoring tumor cells by PA and fluorescent images. AuNPs behave as fluorescent quenchers, PTAs, and cell monitoring by PA images. MMP sensible peptides linked to BSA drive the nanoplatform to MMPs’ overexpressing cells. *Abbreviations: ACPPs, activable cell-penetrating peptides; DOX*, *doxorubicin; MMP, matrix metalloproteinase; NM, nanomaterial; NPs, nanoparticles; PA, photoacoustic; PDT, photodynamic therapy; PTAs, photothermal transduction agents; PSs, photosensitizers; PTT, photothermal therapy.*

However, chemotherapy affects only cells at the tumor edges due to the complexity of the tumor tissue itself. Hence, in order to penetrate deep into the tumor tissue, improve cell uptake, intracellular delivery, and timely drug discharge, multistage drug delivery systems that respond to TME stimuli such as acidic pH, redox environment, hypoxia, and overexpression of proteases like MMPs had to be developed (Chen et al., 2017). In this context, MMP degradable peptides or MMPs substrates (gelatin or collagen) have also been included in the construction of drug nanocarriers. Likewise, ACPP and a cell ligand can improve nanocarriers’ delivery. For instance, the nanocarrier AnACNP contains Angiopep-2, which possesses a great affinity to low-density lipoprotein receptor-1 (LPR1) overexpressed in the surface of some cancer cells such as gliomas, and the E8-6-aminohexanoyl-PLGLAG-R8 ACPP (Gao et al., 2014). Furthermore, the PEG-poly(ε-caprolactone) (PEG-PCL) copolymers are functionalized with the ACPP, angiopep-2, and DXT. Therefore, when the nanocarrier is near the glioma cells or tumor tissue, MMP-2 disrupts the PLGLAG peptide allowing the nanocarrier to attach to the cell through the angiopep-2 favoring its internalization (Figure 6A).

An interesting topic is the use of gelatin in the construction of NPs for the delivery of cisplatin (Vaghasiya et al., 2021). During NPs construction, a cisplatin-gelatin suspension is prepared, and after de-solvation and crosslinking processes, cisplatin-gelatin (CG) NPs are obtained. The CG-NPs are decorated with concanavalin-A (con-A) (CCG-NPs) that can bind to mannose receptors at the surface of cancer cells. Control release of cisplatin was evaluated by incubating the CCG-NPs with MMP-2. Besides, CCG-NP cell attachment and uptake were assayed on lung adenocarcinoma A549 cells in which NPs were encapsulated with FITC. When CCG-NPs are added to A549 cells, MMP-2, and MMP-9 in the cell medium degrade the gelatin matrix, and NPs bind to the cells’ surfaces. Once NPs are internalized, cisplatin is released, inducing a reduction of cell viability.

On the other hand, most of the NMs that constitute the nanocarriers are organic, but some inorganic NPs have also been used, and the peptides included in their construction do not affect their physicochemical characteristics until the nanosystem reaches the target tumor (Yao et al., 2018). In the tumor, when MMPs cleave their corresponding substrate, NMs can undergo changes in their properties (size, charge, hydrophobicity/hydrophilicity, zeta potential, and structure) which lead to NMs’ cell internalization, drug release, the manifestation of characteristics inherent to the NP or the groups associated with it (for example fluorescence emission), dissolution and cell elimination. For instance, MMP sensible peptide-crosslinked nanogels (pNGs) were designed to deliver doxorubicin (DOX) (Nagel et al., 2020). The pNG-DOX complex contains the hyperbranched polymer dendritic polyglycerol (dPG) crosslinked with the fluorogenic peptide Mca-K-([OEG]8-N3-PLGLK[Dnp]ARK-OEG]8-N3)-NH2 (Mca: 7-methoxycoumarinyl-4-acetic acid; Dnp: 2,4-dinitrophenyl; EG: ethylene glycol), with the MMP-7 cleavable sequence. The dPG is conjugated with DOX through a pH-sensitive peptide. When the nanocarrier arrives at the tumor, MMP-7 splits the peptide with the disaggregation of the nanogel, and the fluorescence emitted by the Mca dye is equivalent to MMP-7 enzymatic activity (Figure 6B). Moreover, as an MMP-7 activity effect, the nanocarrier size decreases from ∼200 nm to 40–50 nm facilitating neoplastic cells’ uptake of the pNG-DOX fragments, and DOX is released inside the cell due to the acidic pH. The indocarbocyanine dye (ICC) was used as a fluorescent label for the pNG and their fragments, allowing their distribution monitoring. Furthermore, tumor penetration assays were performed in a 3D model consisting of multicellular tumor spheroids (MCTS) obtained by the co-cultures of cervical cancer HeLa cells and human dermal fibroblasts. The therapeutic effect of DOX was evaluated with cell viability assays in which ATP content was reduced to 22% in MCTS incubated with fragments of pNG-DOX compared with controls.

Similarly, DGL/DOX@PP is a nanoplatform in which DOX is bound through an acidic pH-sensitive hydrazone link to dendrigraft poly-L-lysine (DGL). Then, the DGL-DOX is conjugated to poly(ethylene glycol)-poly(caprolactone) (PP) micelles by the MMP-2 sensitive peptide EGPLGVRGK (@) (Cun et al., 2018). The DGL/DOX@PP size is ∼103 nm, and when incubated with MMP-2, DGL/DOX fragments with a size of ∼30 nm are released. The evaluation of tumor penetration, cellular uptake, and DOX cytotoxicity was done in breast cancer 4T1 cells and 4T1-MCTS. Moreover, to corroborate tumor penetration of the switchable NPs, the DGL/Cy5.5@PP nanoprobe and other formulations were intravenously injected into 4T1 tumor-bearing mice. Higher distribution and deeper penetration in tissue from animals injected with DGL/Cy5.5@PP were found. Firstly, because of its large size that allows its accumulation in tumor tissue due to the EPR effect, and secondly, the release of small fragments by the action of MMP-2 favors cell uptake.

Likewise, a siRNA micellar complex was created to target human programmed death-ligand 1 (PD-L1) implicated in anti-apoptotic signals and reduced T cell recognition of neoplastic cells (Wen et al., 2021). A nanocarrier comprised of polyethyleneimine (PEI) polymers coupled to hyaluronic acid (HA) and the MMP-2 sensitive peptide GPLGLAGC was designed to deliver PD-L1-siRNA to cancer tissue. HA was used to form a protective hydrophilic surface contributing to the EPR effect. Additionally, the nanocarrier HA-P-PEI/siRNA size was ∼186 nm, and after the exposure to MMP-2, the resulting fragments had a size <10 nm, suitable for cellular uptake. Furthermore, cellular internalization was confirmed in human lung adenocarcinoma NCI-H1975 cells by incubating HA-P-PEI/siRNA with Cy3-siRNA that emits red fluorescence. Moreover, NCI-H1975 tumor spheroids incubated with HA-P-PEI/siRNA demonstrated a deep tumor penetration due to the small size of the fragments released to the culture medium after MMP-2 activity and the PEI positive charge. In addition, RT-PCR and Western blot analysis showed a decrease in PD-L1 expression, corroborating the effectiveness of this nanocarrier.

Additionally, nanocarriers that include MMP-2 sensitive peptides conjugated with a molecule for PDT have been developed. Briefly, PDT consists in the internalization of a photosensitizer (PS) into the tumor tissue followed by light irradiation (Kim et al., 2020). PS in the excited state transfers electrons to O_2_ molecules producing superoxide anion radical and then a cascade of ROS (Kwiatkowski et al., 2018). However, TME has hypoxic conditions that may interfere with PDT. This circumstance can be overcome with PTT, which elevates the tissue temperature (see below) (Liu Y. et al., 2019).

For instance, a nanocarrier used for PDT was constructed with ALA as a prodrug since it is the precursor of the PS protoporphyrin IX (PpIX) (Wu et al., 2017). ALA was joined through a pH-sensitive hydrazone bond to AuNPs conjugated to ACPP R8PLGLAGEK10 that contained the PLGLAG MMP-2 sensitive peptide. When the nanocarrier reaches the TME, MMP-2 cuts the peptide, and the AuNP-ALA penetrates the cell where ALA is released because of the intracellular acidic pH, and then it is transformed into PpIX, causing photodynamic cytotoxicity (Figure 6C).

An alternative therapeutic approach used for cancer control is PTT. PTT employs NPs that absorb and transform NIR light into heat to ablate neoplastic cells with temporal and spatial precision reducing toxic effects (Zhao et al., 2021). Moreover, NPs as photothermal transduction agents (PTAs) have high photothermal conversion efficiency (PCE) and possess the ability to increase the EPR effect enhancing the tumor vascular permeability and decreasing the resistance created by components of the ECM, allowing the PTAs accumulation in the tumor tissue. Furthermore, PTT favors blood flow and increases O_2_ availability in the hypoxic areas (Liu Y. et al., 2019). In addition, the elevation in temperature augments local collagenase activity or favors the collagenase enzymatic activity conjugated to the PTA (Liu Y. et al., 2019). Likewise, NPs used in PTT can destroy nuclear DNA from neoplastic cells inducing apoptosis and inhibiting the DNA repair process, and reverse tumor multidrug resistance by downregulating the expression of multidrug resistance-associated protein 1 (MRP1) (Yu et al., 2021).

An example of a nanocarrier used in PTT is the dual-stimuli synergistical nanosystem for imaging guiding PTT, which uses gold nanorods (AuNRs) linked by the MMP sensitive peptide (Pep) GPLGVRGC to an asymmetric cyanine (Acy) (Zhao et al., 2017). In this nanoprobe, asymmetric cyanine is employed as an image fluorescent and auxiliary photothermal agent sensible to acidic pH, while AuNRs are employed because of their photothermal and fluorescence quencher properties. Furthermore, to improve biocompatibility and tumor targeting, the nanosystem was functionalized with glucosamine (Glu) to form the Pep-Acy/Glu@AuNRs nanoprobe (Figure 6D). Assays performed in murine squamous cell carcinoma SCC7 cells that express MMP-2, MMP-9, and MMP-13 were used to demonstrate nanoprobe cell internalization due to the peptide disruption by MMPs and GLUT receptor overexpression in the cells’ surface. Moreover, activation of fluorescence signal was obtained because of the acidic pH in the cell. In addition, SCC7 tumor-bearing mice were injected with the Pep-Acy/Glu@AuNRs, and after 4 h, animals were irradiated with an 808 nm laser light-guided by the *in vivo* fluorescence images. Likewise, an increase in the temperature up to 50°C was observed using a thermal infrared camera. These experiments showed that the tumors in treated animals were suppressed without recurrence (Zhao et al., 2017).

Similarly, a nanosystem that includes MSNs coated IONPs (Fe_3_O_4_@MSNs) has been developed to deliver DOX and monitor treatment response and nanoprobe accumulation in the tumor tissue by fluorescence and MR images (Li et al., 2018). To target the NPs to the tumor, the MMP-2 sensitive peptide GGPLGVRGK is covalently attached to the NPs’ surface (peptide-Fe_3_O_4_@MSNs) while the MSNs provide DOX encapsulation and an increase in the nanocarrier biocompatibility. Moreover, besides its cytotoxic capacity, DOX emits red fluorescence under 480 nm light excitation. Peptide-Fe_3_O_4_@MSNs incubated with active MMP-2 increases fluorescence signal. The DOX fluorescence emitting signal allowed monitoring DOX distribution and cytotoxicity in MMP-2 overexpressing fibrosarcoma HT1080 cells. Additionally, the increase of the EPR effect and magnetic-guided targeting provided by the Fe_3_O_4_ core properties was examined in HT1080 tumor-bearing mice injected with the peptide-Fe_3_O_4_@MSNs after magnetic treatment. Real-time MR images using Fe_3_O_4_ as a contrast agent showed tumor accumulation of the nanoprobe 3 h after the magnetic treatment. Furthermore, animals injected with the peptide-Fe_3_O_4_@MSNs with magnetic treatment had the smallest tumor size (Figure 6E).

Another nanosystem created for nanotheranostic purposes that require the participation of active MMPs is the GNS@BSA/I-MMP-2 (Xia et al., 2019). This nanoprobe is constituted by gold nanostars (GNS) used for PA imaging and as PTA because of its large PCE, and IR-780 iodide (I), a NIR dye that emits fluorescence and has photothermal and photodynamic effects under NIR laser irradiation. BSA stabilizes the system that enhances nanoprobe biocompatibility, coats the GNS NPs, encapsulates the IR-780, and provides functional groups for the MMP-2 sensitive peptide GPLGIAGQ conjugation (Figure 6F). In this system, the MMP-2 substrate peptide is employed to direct the nanoprobe to neoplastic cells. GNS@BSA/I-MMP-2 cellular uptake and cytotoxic effects were examined in lung adenocarcinoma A549 cells taking advantage of the fluorescence signal emitted by IR-780. Drug targeting and effects of the PTT and PDT were visualized by fluorescence, photothermal and PA images in A549 tumor-bearing mice. Results demonstrated a reduced tumor size in those animals injected with the GNS@BSA/I-MMP-2 nanoprobe.

Likewise, the nanosystem DOX@AuNCs, which includes the MMP-2 sensitive CRVGLPDC peptide and AuNCs functionalized to DOX through a pH-sensitive hydrazone bond, has been developed (Mao et al., 2018). The advantage of using AuNCs instead of AuNPs is that NCs can be conjugated with more significant amounts of DOX, and because of their size, their accumulation in the tumor tissue is high due to the EPR effect. Furthermore, conjugation with a peptide sensitive to MMP-2 allows nanoprobe dissociation into small particles easily uptaken by the neoplastic cell. Moreover, experiments performed in lung cancer A549 tumor-bearing mice injected with DOX@AuNCs demonstrated a decrease in tumor size compared with control groups. In addition, nanoprobe distribution and DOX effects in tumor tissue were monitored by micro-CT images through the injection of Gd conjugated to AuNCs (Gd@AuNCs). In this context, AuNCs improved the CT signal of Gd since AuNCs can act as contrast agents.

### Matrix Metalloproteinases as Nanomedicine Targets

MMPs play an important role in cancer progression, and different strategies have been developed to inhibit their expression and enzymatic activity including synthetic MMP inhibitors (sMMPIs). sMMPIs can be grouped into peptidomimetic, non-peptidomimetic, chemically modified tetracyclines, and thiirane-based slow inhibitors (Li et al., 2013; Yang et al., 2016; Marusak et al., 2016; Meisel and Chang, 2017). Some examples of the former are displayed in Table 4. Likewise, other molecules that show an inhibitory effect on MMPs’ enzymatic activity are sulfamides and sulfonamide derivatives, pyrophosphate (PPi) analogs (bisphosphonates) such as zoledronic acid, the non-steroid drug letrozole, and natural MMP inhibitors (soy isoflavone [Genistein] and the shark cartilage extract Neovastat AE941) (Table 4) (Tauro et al., 2014; Piperigkou et al., 2018; Gonzalez-Avila et al., 2019).

**TABLE 4:** Table MMPs’ inhibitors used in cancer treatment.

MMPI type	MMPI	MMP	Cancer application	Side effects
Peptidomimetic inhibitors	Marimastat (BB-2516) (hydroxamate)	MMP-1 MMP-2 MMP-7 MMP-9 MMP-14	Breast, lung and pancreatic cancer	MSS, GID
	Batimastat (BB-94) (hydroxamate)	MMP-1 MMP-2 MMP-3 MMP-7 MMP-9	Malignant ascites, malignant effusion	MSS, GID
Non-peptidomimetic inhibitors	CGS-27023A (MMI-270)	MMP-1 MMP-2 MMP-3 MMP-8 MMP-9	Advanced colorectal cancer, NSCLC	Arthralgias, myalgias, skin rashes
	Rebimastat (BMS-275291)	MMP-1 MMP-2 MMP-8 MMP-14	NSCLC, breast, prostate, HIV-related Kaposi’s sarcoma	Hypersensitivity, dermatologic events, myalgias, muscle, inflammation
	Tanomastat (Bay 12–9576	MMP-2 MMP-3 MMP-8 MMP-9 MMP-13 MMP-14	Pancreas, ovarian, lung cancer	GID, thrombocytopenia, anemia, electrolyte abnormalities, hyperbilirubinemia
	Prinomastat (AG3340)	MMP-2 MMP-3 MMP-7 MMP-9 MMP-13 MMP-14	NSCLC, prostate, glioblastoma, esophageal, breast, melanoma	Musculoskeletal, hematologic, GID, venous thromboembolism
Chemical modified tetracyclines	Metastat (CMT3, COL-3	MMP-1 MMP-2 MMP-8 MMP-9 MMP-13	HIV-related Kaposi’s sarcoma, brain and central nervous system tumors, prostate	Disease stabilization but non-response
	Minocin (minocycline)	MMP-2 MMP-9	Glioma, prostate	NA
	Periostat (doxycycline)	MMP-1 MMP-2 MMP-8 MMP-9	T-cell, Hodgkin, and B-cell lymphomas, breast, renal cell carcinoma	NA
Thiirane-based slow inhibitors	SB-3CT (compound 40)	MMP-2 MMP-9	T-cell lymphoma and prostate cancer models	NA
	ND-322	MMP-2 MMP-14	Melanoma *in vitro* assays	NA
Small sMMPIs	S3304 (sulfonamide derivative)	MMP-2 MMP-9	Solid tumors, NSCLC	NA
	Disulfiram (Antabuse) (sulfonamide)	MMP-2 MMP-9	NSCLC, pancreatic, glioblastoma, melanoma, prostate, refractory solid tumors	NA
Off-target MMPI	Zoledronic acid (bisphosphonate)	MMP-2 MMP-9 MMP-14 MMP-15	Breast	NA
	Letrozole (non-steroidal hormone	MMP-2, MMP-9	Breast	NA
Natural MMPI	Neovastat AE941 (shark cartilage extract)	MMP-1 MMP-2 MMP-7 MMP-9 MMP-12 MMP-13	Refractory multiple myeloma, colorectal, breast, renal cancer carcinoma	NA, non-response
	Genistein (soya isoflavone)	MMP-2 MMP-9	Breast, pancreatic, prostate	NA
Monoclonal antibodies	Single-chain fragment variables	MMP-1 MMP-2 MMP-3	Breast	NA
	Andecaliximab (GS-5745)	MMP-9	Gastric, breast, pancreatic, colorectal, esophageal, NSCLC	Nausea, pain, neutropenia, GID
	AB0041, AB0046	MMP-9	Colorectal	NA
	DX-2400	MMP-14	Fibrosarcoma, breast, melanoma	NA

GID, gastrointestinal disorders; MMP, matrix metalloproteinase; MSS, musculoskeletal syndrome; NA, not available; NSCLC, non-small cell lung cancer.

sMMPIs have been used alone or combined with chemotherapy or immunotherapy in cancer treatment; however, several side effects have been reported. For example, marimastat chelates the zinc ion of the MMPs catalytic site but might also inhibit the activity of other proteases that require zinc and calcium, such as ADAM-10 and ADAM-17 also known as TNF-α converting enzyme (TACE) (Winer et al., 2018). Furthermore, the inhibition of ADAM-10 and ADAM-17 has been associated with the musculoskeletal syndrome characterized by myalgias, tendinitis, and arthralgias. This syndrome is associated with the inhibition of ADAM-10 and ADAM-17 and its ability to activate TNF-α pro-form and the degradation of TNF-α receptor II (TNF-RII) by ADAM-17 (Winer et al., 2018). Furthermore, patients treated with marimastat can also develop fibrosis due to the inhibition of MMP-1. Unfortunately, using other sMMPIs with more specific MMP targets was ineffective since they had a poor effect on cancer survival and produced serious side effects (Table 4) (Piperigkou et al., 2018; Winer et al., 2018). In this context, some monoclonal antibodies targeting MMPs’ catalytic sites have been developed to avoid sMMPIs non-specificity and side effects (Table 4) (Piperigkou et al., 2018; Winer et al., 2018).

**FIGURE 7:**
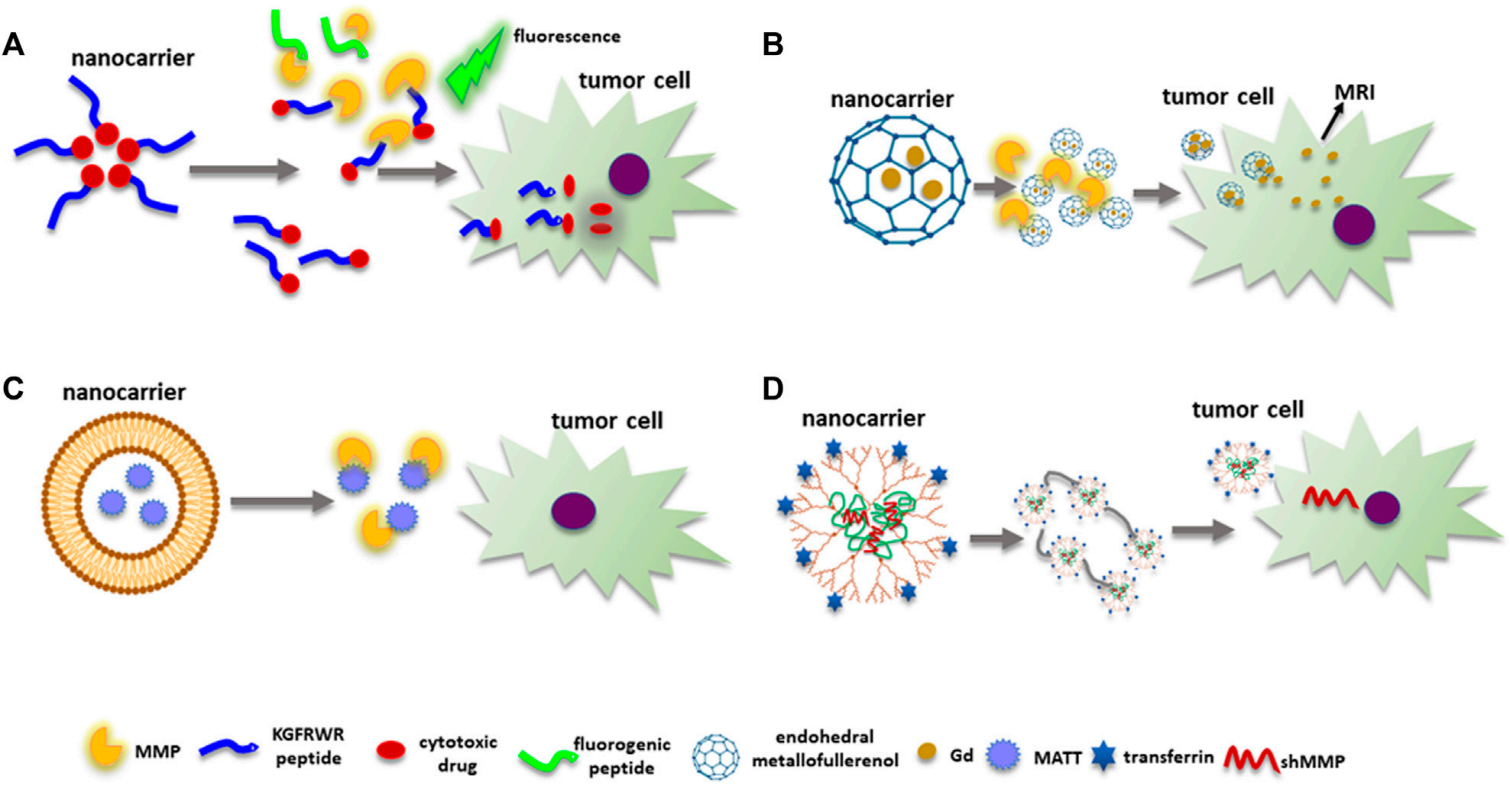
MMPs as targets for nanomedicine. **(A)** The KGFRWR peptide and DOX form a nanofiber. The peptide inhibits MMPs’ enzymatic activity, while DOX has cytotoxic effects. An MMP substrate with fluorogenic capacities is also used to quantify MMPs enzymatic activity. **(B)** Metallofullerenol NPs allosterically inhibit MMPs’ enzymatic activity. Endohedral Gd@C82(OH)22 carries Gd inside to avoid toxic effects allowing MRI images. **(C)** Nanocarriers can deliver sMMPIs such as MATT next to the neoplastic cells avoiding toxic side effects. **(D)** Genetic material to inhibit MMPs’ expression can be delivered by nanocarriers with specific tumor targeting molecules such as transferrin. *Abbreviations: MATT, marimastat; sMMPIs, MMPs’ synthetic inhibitors; MMP, matrix metalloproteinase.*

On the other hand, the nanotechnological approach has been used to inhibit MMPs’ expression and enzymatic activity, avoiding sMMPIs side effects. In this context, some NMs directly inhibit MMPs’ synthesis and enzymatic activity. For instance, a nanofiber system consisting of DOX linked to the KGFRWR peptide (an amyloid *ß* protein derivate) was used to decrease tumor growth and lung metastasis in a hepatocellular carcinoma SMMC7721 tumor-bearing mice model (Ji et al., 2018). The effect on cancer progression was due to DOX cytotoxicity and KGFRWR peptide inhibition of MMP-2 enzymatic activity. Moreover, MMPs fluorogenic peptide MOCAC-PLGLDap(Dnp)AR-NH2, an MMP substrate, was incubated with MMP-2 in the presence of DOX-KGFRWR for 2 h. Results demonstrated a half maximum inhibitory concentration (IC_50_) value of 5.7 × 10^–6^ M for DOX- KGFRWR, while the reported IC_50_ value for batimastat is 4 × 10^–9^ M (Figure 7A).

Similarly, the metallofullerenol Gd@C_82_(OH)_22_ nanoparticles can block MMP-2 and MMP-9 synthesis and activity (Figure 7B) (Kang et al., 2012). Moreover, Gd@C_82_(OH)_22_ binds to amino acid residues next to the S’ pocket of MMP-9 but not to the catalytic site, causing an allosteric inhibition of substrate degradation. Moreover, a decrease in tumor growth and angiogenesis was observed in a JF305 pancreatic carcinoma model injected with Gd@C_82_(OH)_22_ by MRI since the nanosystem can be used as a contrast agent.

Interestingly, the use of nanocarriers to transport sMMPIs to tumor tissue has been studied in an attempt to reduce side effects, for instance, lysolipid-containing thermosensitive liposomes (LTSLs) to deliver marimastat (MATT) (Figure 7C) (Lyu et al., 2019). The MATT-LTSL nanosystem was injected into 4T1 tumor-bearing mice to evaluate its effects on tumor growth and biodistribution. Results showed a decrease in tumor size, angiogenesis, and lung metastasis when mice were injected with the nanosystem and after hyperthermia treatment, due to the reduction in the enzymatic activity of MMP-2 and MMP-9 caused by MATT. However, despite the nanosystem effects on tumor growth and metastasis, MATT has no cytotoxic impact on neoplastic cells. Therefore, using a nanoprobe like this with a chemotherapeutic drug was suggested.

Another novel approach has been the design of more specific nanocarriers to deliver genetic material to inhibit MMPs’ expression. For example, peptide nanofibers (PNFs) conjugated with siMMP-2 (PNF:siMMP-2) were used in a glioblastoma U-87 MG cells invasion assay (Mazza et al., 2019). Results showed that cell migration inhibition was similar when PNF:siMMP-2 was used, and lipofectamine:siMMP-2 was employed as a positive control.

Similarly, a platform to deliver shMMP-9 plasmid was designed and consisted of biodegradable poly(L-lysine) dendrons (PLLD) conjugated to chitosan (CS) that improves the delivery of shMMP-9 (Figure 7D) (Liu et al., 2018). PLLD-CS was functionalized with transferrin (Tf) to target nasopharyngeal carcinoma HNE-1 cells since they overexpress the Tf receptor (TFRC1). Transfection of the PLLD-CS-Tf/pMMP-9 complex to HNE-1 cells showed a higher diminution of MMP-9 expression than cells transfected with complex without Tf (CS-PLLD/pMMP-9). Moreover, induction of apoptosis and a decrease in invasion capacity were observed in cells transfected with the CS-PLLD-Tf/pMMP-9 probe. Furthermore, mice bearing HNE-1 tumors treated with the CS-PLLD-Tf/pMMP-9 complex showed a 77% decrease in tumor growth in comparison with treatment with other formulations 21 days after injection of the complex with non-toxic effects observed in main organs such as the brain, kidney, heart, spleen, lung, and liver. Interestingly, a lower fluorescence signal was observed in these organs, with the higher signal seen in tumors when animals were treated with CS-PLLD-Tf/pMMP-9 compared to CS-PLLD/pMMP-9 in which the green fluorescence signal was higher, particularly in lung and kidney. This finding demonstrates the relevance of including a target ligand when constructing a nanocarrier.

Additionally, nanoplatforms for the delivery of siRNA MMP-9 plasmid and docetaxel (DOC) were created (Zhou et al., 2016). A star-shaped copolymer formed by amphiphilic octadecane (C18)-modified hyperbranched polyglycerol (HPG) (HPG-C18) that encapsulates DOC conjugated with PLLD, which interacts with the siRNA MMP-9 plasmid (HPG-C18-PLLD/DOC/MMP-9 complex) was tested. Breast cancer MCF-7 cells were incubated with the HPG-C18-PLLD/MMP-9 complex causing a significant reduction in MMP-9 expression. The transfection efficiency was due to PLLD and HPG-CD18 hyperbranched structure. In addition, an increase in apoptosis was observed in cells incubated with HPG-C18-PLLD/DOC/MMP-9 complex compared to those that received HPG-C18-PLLD/DOC and HPG-C18-PLLD/MMP-9, demonstrating that the co-delivery of siRNA MMP-9 and DOC enhanced apoptosis. Moreover, the smallest size and slowest tumor growth rate were observed in tumors from mice bearing MCF-7 tumors injected with HPG-C18-PLLD/DOC/MMP-9 complex.

Similarly, a nanoplatform consisting of the conjugation of the cationic hyperbranched poly(amido amine) (HPAA) with MTX and shMMP-9 plasmid (HPAA-MTX/MMP-9) was designed (Tang et al., 2018). Once the nanoprobe is in the cell cytoplasm, HPAA is degraded by glutathione (GSH), abundant in neoplastic cells favoring the release of MTX and shMMP-9 plasmid. In this context, GSH protects tumor cells from damage through its antioxidant effect, maintenance of redox homeostasis, and xenobiotics detoxification (Bansal and Simon, 2018). Furthermore, the GSH detoxification capacity generates resistance to chemotherapeutic drugs favoring cancer progression. With this in mind, to avoid resistance to MTX, HPAA was included in the nanocomplex because HPAA degradation induces GHS consumption, improving MTX cytotoxic effects. To test HPAA-MTX/MMP-9 effects on cancer cells, *in vitro* and *in vivo* assays were conducted. Breast cancer MCF7 cells were transfected with HPAA-MTX/MMP-9, and results showed a downregulation of MMP-9 expression, reduction of cell invasion and migration capacities, and induction of neoplastic cells’ apoptosis. Moreover, results obtained in nude mice bearing MCF7 tumors demonstrated that treatment with HPAA-MTX/MMP-9 has better effects on reducing tumor growth than other formulations. Further, toxic effects were absent in the lung, heart, kidney, liver, and spleen, corroborating the safety of the nanoplatform.

Additionally, a similar nanoplatform was created with Tf conjugation (Liu T. et al., 2019b). This nanosystem consists on hyperbranched poly(aminoamide) (PAA), MTX, shMMP-9 plasmid and Tf (Tf-PAA-MTX/pMMP-9). The incubation of nasopharyngeal carcinoma HNE-1 cells with a medium containing Tf-PAA-MTX/pMMP-9 complex demonstrated induction of apoptosis and decreased tumor invasiveness. In addition, *in vivo* assays showed a reduction in tumor growth.

Likewise, the nanoplatform Tf-HPAA-GO-DOC/pMMP-9 was created using Tf to improve drug delivery and HPAA to release shMMP-9 plasmid and DOC and reduce drug resistance (Liu T. et al., 2019a). Additionally, GO was included in the nanosystem because it can load high amounts of DOC.

On the other hand, nanocarriers may deliver an agent that indirectly affects MMP expression. For example, the nanoprobe constructed with gallic acid (GA), a natural phenolic compound conjugated with AuNPs (GA-AuNPs), can inhibit MMP-9 expression in EGF treated breast cancer MDA-MB-231 cells (Chen et al., 2016). GA-AuNPs inhibit MMP-9 expression by interfering with the EGF/EGFR signaling pathway since GA-AuNPs block the activation of Akt/p65 and ERK/c-Jun, and p300 protein stabilization which is a co-activator of NF*k*B/Ap-1. Furthermore, the downregulation of MMP-9 reduces the migration and invasion capacities of breast cancer MDA-MB-231 cells. These findings indicate that GA-AuNPs affect the EGF/EGFR signaling pathway, but it is unclear if the NPs act on the EGFR.

## Conclusion and Future Considerations

MMPs play a prominent role in cancer progression; therefore, several therapeutic strategies to interfere with their expression and enzymatic activity have been developed. One of them is the generation of sMMPIs, but their poor efficiency in treating cancer patients with advanced stages of the disease and the production of toxic effects have led to consider other alternatives.

On the other hand, nanotechnology upgrades cancer treatment by increasing tumor accessibility to cytotoxic drugs with fewer side effects. Furthermore, the construction of nanoplatforms allows the combination of treatments such as PDT, PTT, and chemotherapy with imaging techniques (fluorescence, SPECT, CT, MRI, PET, and PA) for the localization of tumor tissue and treatment response follow-up in a single delivery system.

Hence, to improve cancer control taking into account the role that MMPs have during all the steps in cancer evolution and the advantages that the nanotechnology applied to medicine offers, several strategies have been formulated like the construction of biosensors and nanocarriers that include MMP sensitive peptides used in the diagnosis, follow-up and treatment of cancer. Moreover, since MMPs are associated with cancer aggressiveness, nanocarriers conjugated with a target ligand to deliver sMMPIs or genetic material to reduce MMPs’ expression and enzymatic activity at the target tumor have been constructed. In this context, it is important to consider the combination of anti-MMP therapy and other cancer treatments in the same nanoplatform to obtain greater benefits for the patient.

Likewise, NPs can induce cytotoxicity in normal cells due to their physicochemical characteristics. Therefore, it is essential to analyze NPs biodistribution, interactions with cells other than the target cell, and effects on molecules such as proteins during their distribution through the body for clinical applications. Furthermore, cell uptake mechanisms, degradation processes, and elimination of the degraded products need to be clearly defined for any specific nanocarrier before it can be safely used in therapeutics.

Finally, although evidently much research in this regard is still needed, the nanotechnological approach can take advantage of MMPs’ properties in the diagnosis and treatment of cancer, improving patients’ quality of life.
